# Long noncoding RNA GAS5 induces abdominal aortic aneurysm formation by promoting smooth muscle apoptosis

**DOI:** 10.7150/thno.34463

**Published:** 2019-07-28

**Authors:** Xiang He, Shifei Wang, Mengsha Li, Lintao Zhong, Hao Zheng, Yili Sun, Yanxian Lai, Xiaoqiang Chen, Guoquan Wei, Xiaoyun Si, Yuan Han, Senlin Huang, Xinzhong Li, Wangjun Liao, Yulin Liao, Jianping Bin

**Affiliations:** 1 Department of Cardiology, State Key Laboratory of Organ Failure Research, Nanfang Hospital, Southern Medical University, Guangzhou, China; 2 Department of Oncology, Nanfang Hospital, Southern Medical University, Guangzhou, China

**Keywords:** GAS5, abdominal aortic aneurysm, Y-box-binding protein 1, microRNA-21, smooth muscle cell apoptosis

## Abstract

**Objective:** Long noncoding RNAs (lncRNAs) may serve as specific targets for the treatment of abdominal aortic aneurysms (AAAs). LncRNA GAS5, functionally associated with smooth muscle cell (SMC) apoptosis and proliferation, is likely involved in AAA formation, but the exact role of GAS5 in AAA is unknown. We thus explored the contribution of GAS5 to SMC-regulated AAA formation and its underlying mechanisms.

**Methods**: Human specimens were used to verify the diverse expression of GAS5 in normal and AAA tissues. The angiotensin II (Ang II)-induced AAA model in ApoE-/- mice and the CaCl_2_-induced AAA model in wild-type C57BL/6 mice were used. RNA pull-down and luciferase reporter gene assays were performed in human aortic SMCs to detect the interaction between GAS5 and its downstream targets of protein or microRNA (miR).

**Results:** GAS5 expression was significantly upregulated in human AAA specimens and two murine AAA models compared to human normal aortas and murine sham-operated controls. GAS5 overexpression induced SMC apoptosis and repressed its proliferation, thereby promoting AAA formation in two murine AAA models. Y-box-binding protein 1 (YBX1) was identified as a direct target of GAS5 while it also formed a positive feedback loop with GAS5 to regulate the downstream target p21. Furthermore, GAS5 acted as a miR-21 sponge to release phosphatase and tensin homolog from repression, which blocked the activation and phosphorylation of Akt to inhibit proliferation and promote apoptosis in SMCs.

**Conclusion:** The LncRNA GAS5 contributes to SMC survival during AAA formation. Thus, GAS5 might serve as a novel target against AAA.

## Introduction

Smooth muscle cell (SMC) apoptosis is an important pathological feature that leads to various mechanisms regulating abdominal aortic aneurysm (AAA) 1, a cardiovascular disease that can lead to fatal rupture 2-7. Many studies have focused on the underlying mechanism of SMC apoptosis for its potential value in the diagnosis and treatment of AAA. The majority of these studies suggested that various proteins can be used as biomarkers and therapeutic targets in AAA diagnosis and treatment because of their role in the regulation of SMC apoptosis 4-7. In addition, evidence suggests that microRNA (miR)-21 may be a crucial protective factor that can be targeted to inhibit SMC proliferation and apoptosis, thereby preventing AAA formation 2. However, traditional methods of administering anti-protein or anti-miR-21 agents have limited applications due to their systemic effects on other organ systems, which are often affected to a greater extent than the aorta 8. Long noncoding RNAs (lncRNAs), a type of noncoding RNA transcript longer than 200 nucleotides, are often expressed in specific locations, thereby displaying the potential to target specific tissues/cells rather than having systemic effects as conventional treatments. LncRNAs have emerged as key components of the address code and can function as miRNA host transcripts, protein scaffolds and mRNA effectors 9, 10. The aberrant expression or mutation of many lncRNA genes has been implicated in various human diseases 10. Recently, multiple lncRNAs have been suggested to contribute to SMC apoptosis during AAA formation 11, 12, among which lncRNA H19 has been demonstrated to participate in AAA development through its regulation of SMC survival 13, 14. Nonetheless, the roles of other lncRNAs in AAA and their therapeutic potential against AAA formation remain elusive.

LncRNA GAS5 is functionally associated with several biological processes, including cell proliferation, apoptosis, differentiation, and growth arrest 15, 16. Recently, GAS5 has been identified as a critical regulator that can rescue the proliferative/migratory phenotype of vascular SMCs 17, 18. Many studies have reported that GAS5 acts as a protein scaffold to regulate the downstream targets of p21^19^, ^20^ and phosphatase and tensin homolog (PTEN) 21, 22, which are validated targets associated with SMC proliferation and apoptosis during aneurysm formation. Moreover, studies have reported that GAS5 acts as a “miRNA sponge” to regulate miR-21 expression in many other diseases, including cancer 15, osteoarthritis 23, and cardiac fibrosis 21. MiR-21 is one of the most commonly and significantly deregulated miRNAs in several cardiovascular diseases 24 and is the only miRNA that has been demonstrated to play a central role in AAA formation through the regulation of SMC proliferation and apoptosis 2. Therefore, we hypothesized that GAS5 stimulates SMC apoptosis and proliferation via simultaneously regulating both proteins and miR-21 and ultimately contributes to AAA formation.

In this study, we used angiotensin (Ang) II- and CaCl_2_-induced AAA mouse models to investigate the role of GAS5 in AAA formation and its underlying mechanism. GAS5 is significantly upregulated in SMCs in the presence of angiotensin II (Ang II) and in the aortas of human and mouse AAA models. GAS5 overexpression represses cell proliferation, induces SMC apoptosis and accelerates AAA formation in mouse models. GAS5 acts as a sponge of miR-21 to release PTEN from miR-21-mediated suppression, thereby inhibiting the phosphorylation and activation of Akt. Additionally, GAS5 and Y-box-binding protein 1 (YBX1) form a positive feedback loop to promote downstream p21 expression.

## Materials and Methods

The data that support the findings of this study are available from the corresponding author upon reasonable request.

### Human abdominal aortic aneurysm (AAA) tissue samples

Human AAA samples were obtained according to multi-center clinical research project approved by the Ethics Committees of NanFang Hospital (ethical approval number: NFEC-2019-086; Table S1), the leading research hospital. AAA samples and adjacent normal aortic tissues were retrospectively collected from patients who had undergone AAA resection surgery and had the resected tissues been stored in biological database. Additionally, this multi-center clinical research project received an informed consent exemption from the ethics committee of the NanFang Hospital (Table S2). The detailed characteristics of patients are available in Table S3. All samples were immediately frozen in liquid nitrogen after collection and stored at -80°C until further processing.

### Mice

All animal protocols were approved by the Animal Research Committee of Southern Medical University and performed in accordance with the Guide for the Care and Use of Laboratory Animals published by the United States National Institutes of Health. The *in vivo* experiments were performed with 10- to 12-week-old male C57BL/6 mice (CaCl_2_ model) and 12- to 16-week-old male *ApoE-/-* mice (Ang II infusion model).

### Angiotensin II infusion model

We used 10- to 12-week-old male C57BL/6J mice and 12- to 16-week-old male ApoE-/- mice. In brief, an osmotic minipump (Alzet, model 2004; DURECT Corporation, Cupertino, CA) was subcutaneously implanted in the dorsum of the neck of an anesthetized mouse via a small incision. Ang II (A9525; Sigma) or normal saline (0.9% NaCl) was infused via the minipump at a rate of 1 μg/kg per minute as previously described 25 for 28 days.

### CaCl2-induced AAA model

C57BL/6J mice were anesthetized by an intraperitoneal injection of pentobarbital (40mg/kg) before being subjected to laparotomy. The abdominal aortic segment below the renal arteries and above the bifurcation of the iliac arteries was isolated from the surrounding retroperitoneal structures. The diameter of this aortic segment was measured in triplicate by video microscopy. Then, a cotton gauze with 0.5 mol/L CaCl_2_ was spread over the external surface of the aortic passage for 15 minutes. A cotton gauze with NaCl (0.9%) was used for the sham operation in control mice. Then, the aorta was rinsed with 0.9% sterile saline, and the incision was sutured. After 3 weeks, the mice were sacrificed and subjected to laparotomy for further assessment.

### Aneurysm quantification

Mice were euthanized, and an abdominal incision was made to detect the presence of aortic aneurysms. To show the aorta, phosphate-buffered saline (PBS, 10 mL) was injected from the left ventricle and overflowed from the incision at the right atrium. The periadventitial tissue was dissected under an anatomical microscope, and the aorta was then photographed. The suprarenal artery was identified as the passage between the last pair of intercostal arteries and the right renal branch.

The maximum outer width of the abdominal aorta or descending part of the thoracic aorta was measured using Image-Pro Plus software to quantify the AAA size (Media Cybernetics). To confirm the occurrence of aneurysm, the established human aneurysm definition was used (>50% increase in the outer aortic diameters compared with those of aortas from saline-infused mice). The definition of aortic rupture was described previously 26.

The methods for measuring the intimal and outer widths of both the descending thoracic aorta and abdominal aorta were described previously 27. AAA evaluation was performed by an investigator who was blinded to the experimental treatments. A second investigator was invited to confirm the aortic diameters and areas of mice under various treatments.

### *In situ* hybridization (ISH)

*In situ* hybridization (ISH) was performed using a Panomics QuantiGene ViewRNA ISH tissue assay system (Affymetrix, Santa Clara, CA) to determine GAS5 expression and distribution in human and mouse aortic tissues. Aortic specimens were fixed in 10% formaldehyde, embedded in paraffin and cut into 5-µm-thick sections. After digestion with proteinase K, the aortic tissue sections were hybridized at 37°C overnight with a custom designed GAS5 probe (5'-tccttggggacacaactgtccataaggtgctatccagagccacactgcat-3' for humans and 5'-aatcattgtgtttgcagtgccttcacttgaggtgacccattaataccttt-3' for mice). Then, the samples were incubated overnight with an anti-digoxin-alkaline phosphatase (AP) Fab fragment. The cytoplasm was stained with NBT/BCIP in the dark, and GAS5 ISH signals were identified as blue-purple speckles.

### TUNEL

After appropriate treatment, human aortic smooth muscle cells (HASMCs) in each experimental group were fixed with paraformaldehyde for 1 hour, and apoptotic cells were labeled using an *in situ* cell death detection kit (TMR red) (Roche, Switzerland). DAPI staining reagent (Beyotime, China) was used to stain nuclei. Six visual fields were selected from each group to calculate the proportion of TUNEL-positive cells. Apoptotic cells, expressed as percentages (cell count with positive TUNEL staining/cell count with positive DAPI staining), were compared and analyzed. Images were collected by fluorescence Leica (TCS Sp8) confocal microscopy.

### Immunohistochemistry (IHC)

Aortic samples were deparaffinized, and endogenous peroxidase activity was blocked with 3% hydrogen peroxide, followed by subsequent incubation with 10% bovine serum to block nonspecific binding sites. Immunohistochemistry was performed as described previously 28, 29. The primary antibodies details are shown in Table S4.

### Ultrasonography for aortic aneurysm

Twenty-eight days after Ang II infusion, the mice that survived were anesthetized with intraperitoneal pentobarbital (40 mg/kg) and subjected to 2-dimensional color-coded Doppler ultrasound imaging utilizing a Sequoia ultrasound system with a linear array ultrasound transducer (15 L8-S; mechanical index, 0.17; frequency, 14 MHz; Siemens Medical Systems).

### Cell culture and treatment

HASMCs were purchased from Cellbio Company (Shanghai) and cultured and propagated in growth medium (Dulbecco's Modified Eagle's Medium) supplemented with 10% fetal bovine serum, streptomycin (100 μg/mL) and penicillin (100 U/mL). Cells were used when they were passed to 3 to 6 generations and grown to 70% to 80% confluence before treatment with different agents. The cells were then serum-starved and treated with small interfering RNA (siRNA)-GAS5, scrambled (SCR) siRNA, pcDNA-GAS5, vector, or PBS at 37°C for 24 hours (the siRNA and human GAS5 sequences are available in Table S5). PBS was used as a negative control. After 24 hours, the negative control cells were treated with PBS, and cells under other treatment conditions were further treated with Ang II. Then, all the cells were incubated at 37°C for 24 hours. After the incubation period, the HASMCs were used for additional experiments.

### Injection of adeno-associated viruses (AAVs)

AAV-GFP, AAV-GFP-GAS5, SCR short hairpin RNA (shRNA), and shRNA-GAS5 were synthesized by GeneChem Co., Ltd. (Shanghai, China). ApoE-/- and C57BL/6J mice were intravenously injected with AAV-GFP, AAV-GFP-GAS5, SCR shRNA, or sh-GAS5 at a dose of 2 × 10^12^ viral genome particles per animal using an insulin syringe and a 30-gauge needle (BD, NJ, USA; the AAV and mouse GAS5 sequences are available in Table S5). The injected mice were processed to construct AAA models after 30 days. The fluorescence intensity of staining with an anti-GFP antibody was used to determine the AAV transfection efficiency.

### Immunofluorescence analysis

For *in vitro*-cultured HASMCs, the medium was removed by washing with PBS. Then, the cells were fixed with 4% polyoxymethylene, permeabilized with 0.2% Triton X-100 and blocked with PBS containing 5% bovine serum albumin. The immunofluorescence analysis was performed as described previously 30, 31. The primary antibody details are available in Table S6.

### RNA fluorescence *in situ* hybridization

HASMCs on coverslips were fixed in 4% paraformaldehyde, washed 3 times with PBS and then permeabilized with 0.2% Triton X-100 in PBS for 30 minutes. The HASMCs were hybridized with a hybridization buffer (RiboBio, Guangzhou, China) and incubated with a labeled GAS5 probe overnight at 37°C. The GAS5 probe was purchased from BersinBio (Guangzhou, China). The cells were then washed with 2× SSC, 1× SSC, and 0.5× SSC and incubated with a mouse anti-digoxin antibody conjugated with AP (Boster Biotechnology Co., Ltd., Wuhan, China). Subsequently, the cells were incubated in DAPI (Beyotime Biotechnology, Shanghai). Images were obtained using aLeica (TCS Sp8) confocal microscope.

### Cell cycle analysis

For the analysis of cell cycle distribution, HASMCs were cultured in 6-well plates for 48 hours. The cells were then treated as previously described (control, Ang II, Ang II+vector, Ang II+pcDNA-GAS5, Ang II+SCR siRNA, and Ang II+si-GAS5). The cells were trypsinized, harvested, washed with cold PBS, fixed with 75% ethanol at 4°C for 2 h and then centrifuged. Next, the cells were washed twice with cold PBS and labeled with propidium iodide by incubation with propidium iodide solution and RNase A at room temperature for 30 minutes in the dark. Cell cycle distribution was analyzed by flow cytometry (Beckman Coulter, CytoFlex, USA) after treatment. All of the experiments were performed in accordance with the instructions of the Cell Cycle Kit (KeyGen Biotech. Co, Ltd, China).

### RNA isolation and quantitative real-time PCR (qRT-PCR)

Total RNA was extracted from HASMCs and aortic tissues from human AAA and adjacent tissue specimens or from different mouse groups using TRIzol reagent (Invitrogen, 15596026) according to the provided protocol. cDNA was reverse transcribed from 1 μg of total RNA using PrimeScript^TM^ RT Master Mix (TaKaRa Biotechnology, Dalian, China). For reverse transcription of GAS5, strand-specific and reverse GAS5 primers were used. Quantitative real-time PCR (qRT-PCR) was performed with the SYBR Premix Ex Taq^TM^ Kit (Takara Biotechnology, Dalian, China) using a Light Cycler 480 II system (Roche Diagnostics, Basel, Switzerland). Glyceraldehyde-3-phosphate dehydrogenase mRNA was used as an internal control to normalize gene expression using the 2-ΔΔCt method. The primer sequences are presented in Table S7.

### Western blot analysis

Briefly, HASMCs and aortic tissues from human AAA and adjacent tissue specimens or from different mouse groups were harvested and lysed in radioimmunoprecipitation assay buffer (Dingguo Changsheng, Beijing, China) containing the protease inhibitor phenylmethanesulfonyl fluoride (Beyotime, P0013B) and phosphatase inhibitors (Beyotime, P1081). Protein concentrations were determined using a BCA Protein Assay Kit (Beyotime, P0010). Western blot was performed as described previously 32, 33. The primary antibodies used are presented in Table S8.

### Pull-down

The probes for GAS5 and its antisense RNA for RNA pull-down and DNA pull-down were designed and synthesized by Gzscbio Co. Ltd. (Guangzhou, China). Isolated HASMCs were washed in PBS, lysed in 0.5 mL of co-immunoprecipitation buffer, and incubated with 3 µg of biotinylated DNA oligo probes against a GAS5 back splice sequence at room temperature for 4 hours. Then, the HASMCs were incubated with streptavidin-coated magnetic beads (Invitrogen, SA10004) for another hour at room temperature. RNase-free bovine serum albumin and yeast tRNA (Sigma, Shanghai, China) were utilized to prevent the nonspecific binding of RNA and protein complexes. RNA complexes bound to beads were extracted by TRIzol for qRT-PCR analysis, and proteins were resolved by sodium dodecyl sulfate polyacrylamide gel electrophoresis and silver stained. The specific bands were excised and analyzed by mass spectrometry.

### Chromatin immunoprecipitation assay

As previously described, chromatin immunoprecipitation assays were performed using the EpiQuik Chromatin Immunoprecipitation Assay Kit (EpiGentek, Brooklyn, NY) according to the manufacturer's protocol. An anti-YBX1 antibody (10 μg) or control IgG antibody (10 μg) was used for immunoprecipitation. Then, qRT-PCR and gel electrophoresis were used to quantify the DNA fragments at the predicted YBX1binding sites.

### RNA immunoprecipitation (RIP)

RNA immunoprecipitation (RIP) experiments were performed with the Magna RIP^TM^ RNA-binding protein Immunoprecipitation Kit (Millipore, Stafford, VA) in accordance with the manufacturer's instructions. An anti-YBX1 antibody (1:20, Proteintech, 20339-1-AP) was used to coimmunoprecipitate RNA. GAS5 expression was measured by qRT-PCR.

### Luciferase reporter assay

GAS5-sv-wt and GAS5-sv-mut were cloned into the luciferase vector psiCHECK-2 (Gzscbio Co. Ltd, Guangzhou, China). For luciferase reporter assays, the miR-21 mimic was cotransfected into HASMCs with the luciferase constructs described above using Lipofectamine 2000 (Invitrogen, Thermo Fisher Scientific). Luciferase activity was measured by the Dual-Luciferase Reporter Assay System (Promega, Madison, WI).

### Statistical analyses

Data were analyzed using SPSS 20.0 software (SPSS Inc., Chicago, IL, USA) and are presented as the mean ± standard deviation (SD). A normal distribution test was performed for all continuous variables. After the confirmation of equal variance among groups, comparisons between two groups were performed with independent-samples *t*-tests, and comparisons among three or more groups were performed with one-way analysis of variance (ANOVA) followed by Bonferroni's tests. Fisher's exact test was applied for the analysis of aneurysm incidence. A *p* value <0.05 indicated statistical significance.

## Results

### GAS5 is significantly unregulated in human and mouse AAA tissues

**Figure 1:**
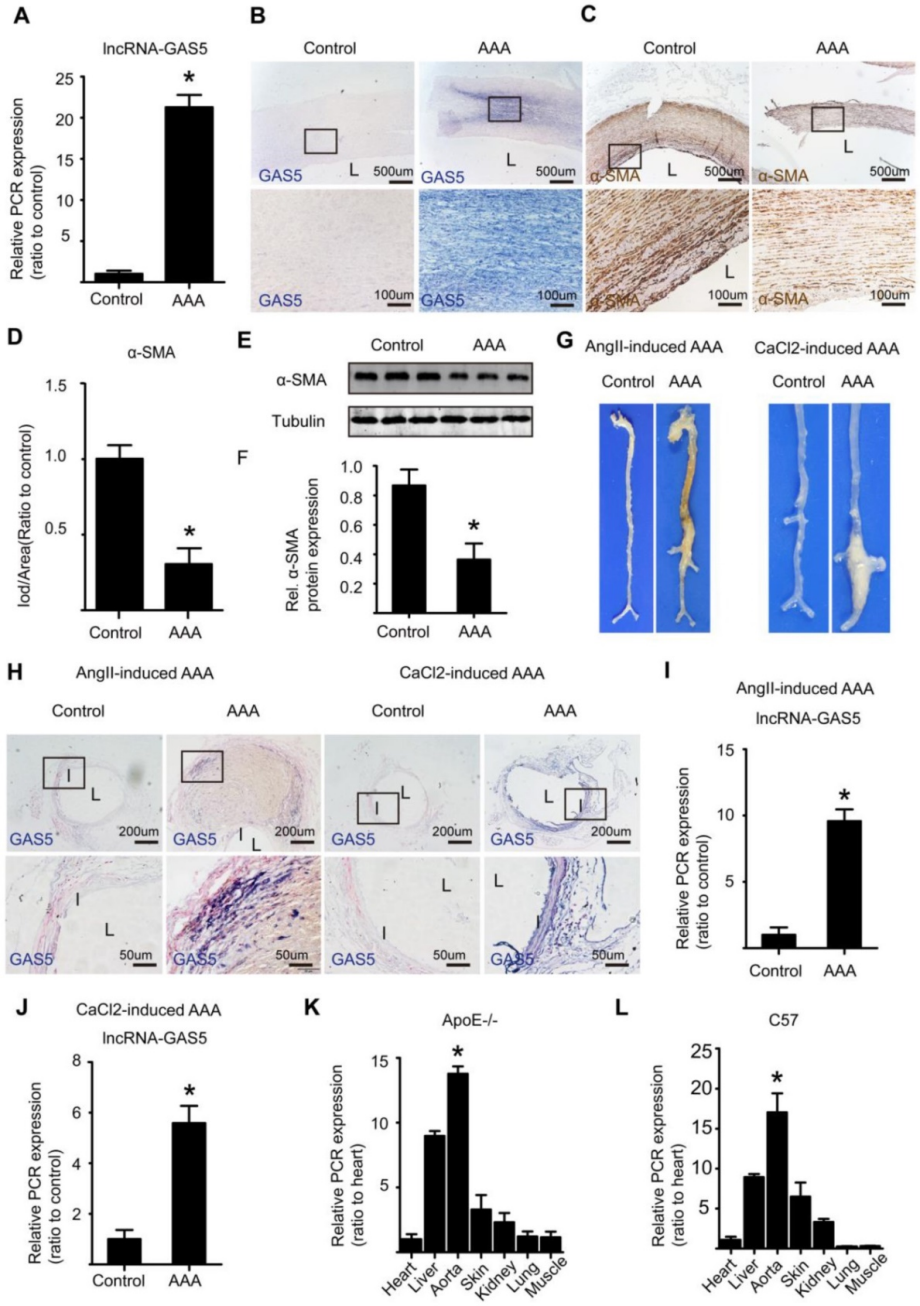
** GAS5 is significantly unregulated in human and mouse AAA tissues.** A. The relative expression of lncRNA GAS5 in human tissue (qPCR). *p < 0.05; n = 10 per group (student's t-test). B. ISH results of GAS5 in human AAA tissue and control tissues (n = 10, bars: upper 500 μm, lower 100 μm, magnified images). C. IHC results of α-SMA in human AAA tissue and control tissues (n = 10**,** bars: upper 500 μm, lower 100 μm, magnified images). D. The relative expression of α-SMA in human AAA tissue and control tissues (IHC). *p < 0.05; n = 10 per group (student's t-test). E. Western blot results of α-SMA in human AAA tissue and control tissues. F. The relative expression of α-SMA in human AAA and control tissues (western blot). *p < 0.05; n = 10 per group (Student's t-test). G. Images depict the characteristics of aortas from ApoE-/- mice treated with Ang II and from C57BL/6J mice treated with CaCl_2_. H. ISH results of aortic GAS5 in Ang II-treated or CaCl2-treated mice (n=5, bars: upper 200 μm, lower 50 μm, magnified images). I. The relative expression of GAS5 in Ang II-induced mouse AAA models (qPCR). *p < 0.05; n = 5 per group (Student's t-test). J. The relative expression of GAS5 in CaCl_2_-induced mouse AAA models (qPCR). *p < 0.05; n = 5 per group (Student's t-test). K. The relative expression of GAS5 in the heart, liver, aorta, skin, kidney, lung, and muscle tissues of ApoE-/- mice. L. The relative expression of GAS5 in the heart, liver, aorta, skin, kidney, lung, and muscle tissues of C57BL/6J mice.

Human AAA and corresponding adjacent normal aortic tissues were retrospectively collected from patients who had underwent AAA resection surgery and had the resected tissues been stored in biological database. IHC results shown that in human normal aortic tissues, MMP2 and MMP9 were almost not expressed while in human AAA tissues, MMP2 and MMP9 were highly expressed, findings that were confirmed by α-SMA expression which was high in human normal aortic tissues while was low in human AAA tissues (Figure S1A-F). Both qPCR (Figure 1A) and ISH (Figure 1B and Figure S2A) showed that the expression of lncRNA GAS5 was substantially higher in human AAA tissues than in the corresponding adjacent normal aortic tissues (p < 0.05) and was accompanied by a significant decrease in the number of HASMCs, as shown by α-SMA IHC (p < 0.05; Figure 1C-D) and western blot (p < 0.05; Figure 1E-F), a crucial pathological process that leads to AAA formation. Interestingly, there was no linear association between GAS5 expression and human AAA diameter (p>0.05; Figure S2B).

As previously described, we established the Ang II-induced mouse AAA model and its corresponding control by slowly infusing Ang II or saline with a minipump for 4 weeks into male ApoE-/-mice. The CaCl_2_-induced mouse AAA model and its corresponding control were established by treating male C57BL/6J mice with CaCl2- or saline-containing gauze for 15 minutes during surgery. As macroscopically observed, mice in both models of AAA displayed more obvious aortic bulges as the corresponding control mice (Figure 1G), showing that we successfully established both types of models. Furthermore, ISH identified significantly higher levels of GAS5 in Ang II- and CaCl2-induced AAA model mice than in control mice (Figure 1H and Figure S2C). Consistent with these results, qPCR also confirmed that the expressions of GAS5 in Ang II- and CaCl2-induced AAA model mice were substantially higher than that in wild-type control mice (p <0.05; Figure 1I-J). Moreover, the aortic expression of GAS5 was the highest among the main organs/tissues of ApoE-/- and C57BL/6J mice (p < 0.05; Figure 1K-L). Then, among various cells of aorta, GAS5 was mainly distributed in the endothelial cells and SMCs, followed by the fibroblasts (Figure S3A). Collectively, these findings suggest a potential role for GAS5 in AAA formation. To determine whether GAS5 is mainly expressed in cytoplasm or nucleus of HASMCs, we firstly performed fluorescence ISH experiments and then isolated the cytoplasmic and nuclear RNA of HASMCs to quantify the expression of GAS5 by qRT-PCR respectively. Both methods found that GAS5 was mainly expressed in the nucleus (Figure S3B-D).

### GAS5 induces apoptosis and represses proliferation in HASMCs

In agreement with the above *in vivo* results, Ang II could also significantly increase GAS5 expression in HASMCs *in vitro* (p < 0.05; Figure S3E). Treatment with Ang II induced apoptosis while decreasing the proliferation rate of HASMCs (Figure S4A-D). However, these effects were attenuated and enhanced by GAS5 knockdown and overexpression, respectively, indicating the regulatory role of GAS5 in HASMC apoptosis and proliferation under conditions similar to AAA formation (Ang II treatment).

**Figure 2:**
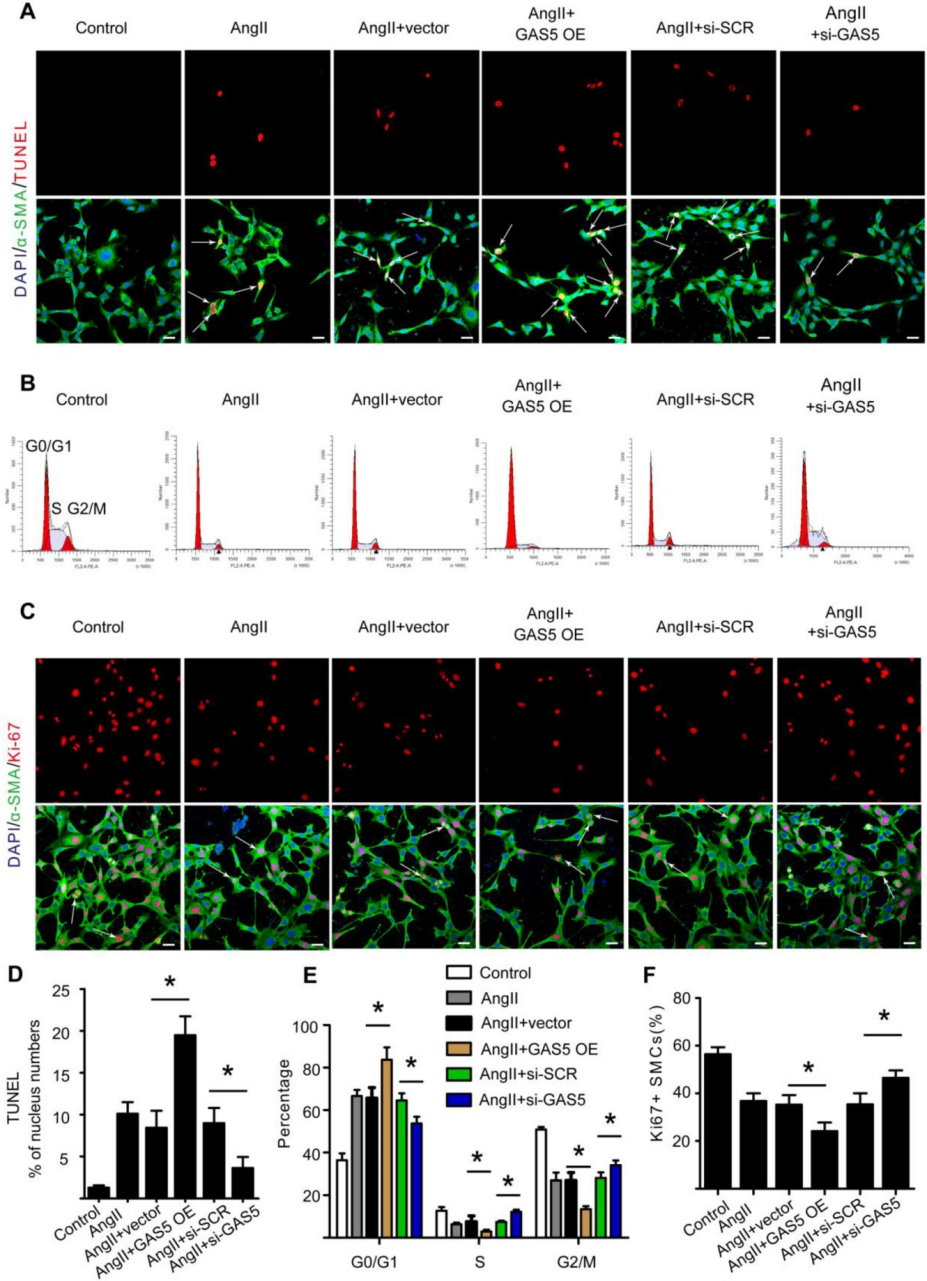
** GAS5 induces apoptosis and represses proliferation in HASMCs.** A. Immunofluorescence staining for DAPI (blue), α-SMA (green) and TUNEL (red) signals after treatment with Ang II, Ang II and vector, Ang II and GAS5 overexpression constructs, Ang II and SCR constructs, or Ang II and GAS5 knockdown constructs for 48 hours (bars, 100 μm). B. The percentage of HASMCs in the G0/Gl phase after HASMCs were treated with Ang II, Ang II and vector, Ang II and GAS5 overexpression constructs, Ang II and SCR constructs, or Ang II and GAS5 knockdown constructs for 48 hours. C. Immunofluorescence staining for DAPI (blue), α-SMA (green), and Ki-67 (red) after treatment with Ang II, Ang II and vector, Ang II and GAS5 overexpression constructs, Ang II and SCR constructs, or Ang II and GAS5 knockdown constructs for 48 hours (bars, 100 μm). D. Quantification of TUNEL-positive HASMCs. *p < 0.05; n=7 per group (one-way ANOVA). E. Quantification of the percentage of HASMCs in the G0/Gl phase. *p < 0.05; n=7 per group (one-way ANOVA). F. Quantification of Ki-67-positive HASMCs. *p < 0.05; n=7 per group (one-way ANOVA).

To further investigate the regulatory effects of GAS5 on SMCs, we designed GAS5 overexpression constructs to enhance GAS5 expression in HASMCs (mediated by pcDNA-GAS5; >1550% promotion) (Figure S4E). Additionally, three different GAS5-targeting siRNAs were designed to reduce GAS5 expression *in vitro*, of which GAS5 siRNA1 (mediated siRNA; > 83% silencing) was the most potent in terms of knockdown efficiency (Figure S4F). In HASMCs, knockdown of GAS5 inhibited apoptosis, as shown by TUNEL (Figure 2A). Simultaneously, knockdown of GAS5 decreased the proportion of cells in the G0/G1 phase (Figure 2B) and increased the proportion of Ki-67-positive HASMCs (Figure 2C). In contrast, overexpression of GAS5 promoted apoptosis (Figure 2A-D) while inhibiting proliferation in HASMCs (Figure 2B, 2E, and 2F).

The current study additionally found that GAS5 involved in SMCs phenotypic conversion, an initial factor for AAA development. Overexpression of GAS5 decreased the markers related to phenotypic conversion, including SMMHC and SM22, while knockdown of GAS5 increased the expression of these markers (Figure S5A-F). Thus, GAS5 may also involve in the initial stage of AAA development.

### Downregulation of GAS5 suppresses Ang II-induced AAA formation in ApoE-/- mice, and overexpression of GAS5 exacerbates AAA development in C57BL/6J mice

Next, we investigated whether GAS5 is involved in mouse AAA development. We used AAVs carrying GAS5 knockdown constructs to inhibit GAS5 expression (sh-GAS5 group) or AAVs carrying GAS5 overexpression constructs (AAV-GFP-GAS5 group) and their corresponding sham control viruses (SCR shRNA and AAV-GFP groups, respectively) to perform gain- and loss-of-function studies on GAS5, respectively. Double immunofluorescence studies confirmed that both GAS5 knockdown and overexpression constructs successfully incorporated into the aortic wall (Figure S6A). Successful overexpression and knockdown of GAS5 in Ang II-induced mouse AAA tissues were further confirmed by qRT-PCR, as the expression of GAS5 was measured in GAS5 knockdown and overexpression construct-transfected mice and compared with that in SCR control-transfected mice (Figure S6B -C). The expressions of GAS5 in other main organs after modulation were shown in Figure S6D-E. Generally, the expression of GAS5 in each organ was changed.

**Figure 3:**
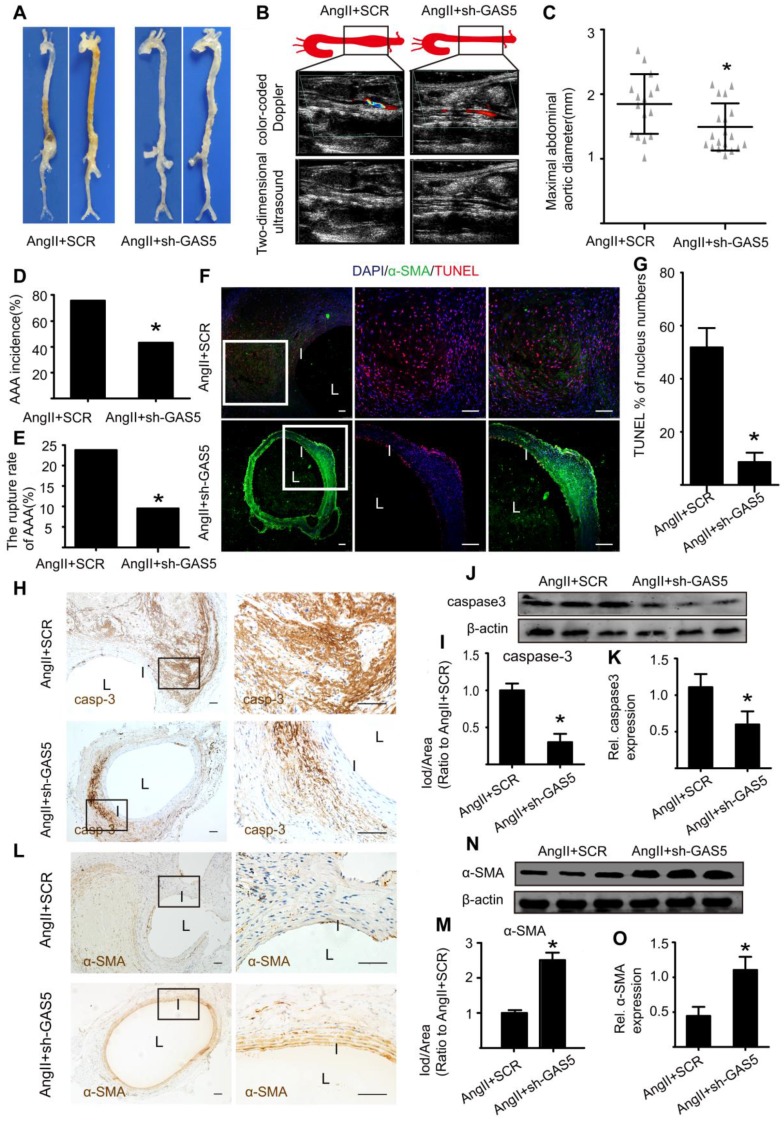
** GAS5 knockdown inhibits aneurysm progression in Ang II-treated ApoE-/- mice.** A. Images depict the characteristics of aortas from ApoE-/- mice treated with Ang II or Ang II and GAS5 knockdown constructs. B. Color-coded doppler and two-dimensional ultrasound imaging of aortas from ApoE-/- mice treated with Ang II or Ang II and GAS5 knockdown constructs. C. Maximal abdominal aortic diameters from ApoE-/- mice treated with Ang II or Ang II and GAS5 knockdown constructs. *p < 0.05; n=21 per group (Student's t-test). D. The incidence of AAA in Ang II-treated ApoE-/- mice. *p < 0.05; n=21 per group (Fisher's exact test). E. The AAA rupture rate of Ang II-treated ApoE-/- mice in control and GAS5 knockdown group. *p < 0.05; n=21 per group (Fisher's exact test). F. Immunofluorescence staining for DAPI (blue), α-SMA (green) and TUNEL (red) signals in aortas from ApoE-/- mice treated with Ang II or Ang II and GAS5 knockdown constructs (bars: left 200 μm, middle and right 100 μm, magnified images). G. Quantification of TUNEL-positive HASMCs.*p <0.05 vs.SCR control; n= 7 per group (Student's t-test). H. IHC of aortic caspase-3 in Ang II-treated ApoE-/- mice (n=7, bars: left 200 μm, right 50 μm, magnified images) when GAS5was knocked down. I. Quantification of caspase-3 protein levels in the aorta (IHC), *p < 0.05; n=7 per group (Student's t-test). J. Caspase-3 protein levels in the aortas of Ang II-treated ApoE-/- mice when GAS5 was knocked down (western blot) (β-actin internal reference). K. Quantification of caspase-3 protein levels in the aortas of Ang II-treated ApoE-/- mice (western blot). *p < 0.05; n=5 per group (Student's t-test). L. IHC of aortic α-SMA in Ang II-treated ApoE-/- mice (n=7, bars: left 200 μm, right 50 μm, magnified images) when GAS5 was knocked down. M. Quantification of α-SMA levels in the aorta (IHC), *p < 0.05; n=7 per group (Student's t-test). N. α-SMA levels in the aortas of Ang II-treated ApoE-/- mice when GAS5 was knocked down (western blot) (β-actin internal reference). O. Quantification of α-SMA levels in the aortas of Ang II-treated ApoE-/- mice (western blot). *p < 0.05; n=5 per group (Student's t-test).

As macroscopically observed, the bulge in the abdominal aorta was significantly more obvious in the control mice than in the sh-GAS5-transfected mice (Figure 3A). Ultrasound imaging also confirmed this finding (Figure 3B). In addition, knockdown of GAS5 reduced the maximal abdominal aortic diameter (Figure 3C), the incidence of AAA formation (Figure 3D) and the rupture rate of AAA (Figure 3E) relative to those in control mice, suggesting that GAS5 knockdown exerts an inhibitory effect on AAA development. We further monitored whether the inhibitory effect of GAS5 knockdown on AAA formation was mediated by the inhibition of SMC apoptosis. Consistent with our hypothesis, knockdown of GAS5 significantly inhibited SMC apoptosis *in vivo*, as identified by TUNEL assays (Figure 3F-G) and caspase-3 protein levels (Figure 3H-K and Figure S7A). Moreover, the expression of α-SMA, an SMC marker, in sh-GAS5-transfected mice was obviously higher than that in control mice (Figure 3L-O and Figure S7B), indicating that more SMCs were preserved in sh-GAS5-transfected mice than in control mice (p < 0.05).

**Figure 4:**
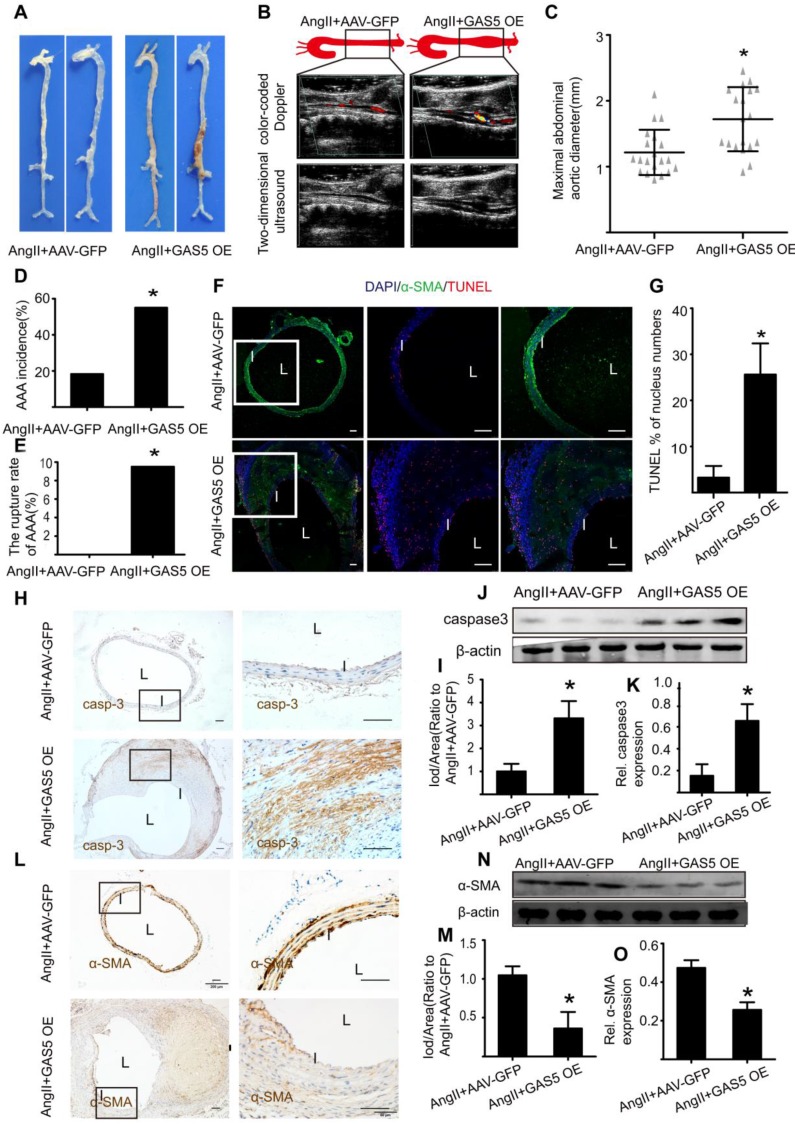
** GAS5 overexpression stimulates aneurysm progression in Ang II-treated C57BL/6J mice.** A. Images depict the characteristics of aortas from C57BL/6J mice treated with Ang II or Ang II with GAS5 overexpression constructs. B. Color-coded doppler and two-dimensional ultrasound imaging of aortas from C57BL/6J mice treated with Ang II or Ang II and GAS5 overexpression constructs. C. Maximal abdominal aortic diameters of C57BL/6J mice treated with Ang II or Ang II with GAS5 overexpression constructs. *p < 0.05; n=21 per group (Student's t-test). D. The incidence of AAA in Ang II-treated C57BL/6J mice. *p < 0.05; n=21 per group (Fisher's exact test). E. The AAA rupture rate of Ang II-treated C57BL/6J mice in control and GAS5 overexpression group. *p < 0.05; n=21 per group (Fisher's exact test). F. Immunofluorescence staining for DAPI (blue), α-SMA (green) and TUNEL (red) signals in aortas of C57BL/6J mice treated with Ang II or Ang II and GAS5 overexpression constructs (bars: left 200 μm, middle and right 100 μm, magnified images). G. Quantification of TUNEL-positive HASMCs. *P<0.05 vs.SCR control; n= 7 per group (Student's t-test). H. IHC of aortic caspase-3 in Ang II-treated C57BL/6J mice (n=7, bars: left 200 μm, right 50 μm, magnified images) when GAS5 was overexpressed. I. Quantification of caspase-3 protein levels in the aorta (IHC), *p < 0.05; n=7 per group (Student's t-test). J. Caspase-3 protein levels in the aortas of Ang II-treated C57BL/6J mice when GAS5 was overexpressed (western blot) (β-actin internal reference). K. Quantification of caspase-3 protein levels in aortas (western blot). *p < 0.05; n=5 per group (Student's t-test). L. IHC of aortic α-SMA in Ang II-treated C57BL/6J mice (n=7, bars: left 200 μm, right 50 μm, magnified images) when GAS5 was overexpressed. M. Quantification of α-SMA levels in the aorta (IHC), *p < 0.05; n=7 per group (Student's t-test). N. α-SMA levels in the aortas of Ang II-treated C57BL/6J mice when GAS5 was overexpressed (western blot) (β-actin internal reference). O. Quantification of α-SMA levels in the aortas (western blot). *p < 0.05; n=5 per group (student's t-test).

In contrast, overexpression of GAS5 resulted in a more obvious bulge in the abdominal aorta relative to that of the control (Figure 4A). Other indicators of AAA severity, such as ultrasound imaging data (Figure 4B), the maximal abdominal aortic diameter (Figure 4C), the incidence of AAA formation (Figure 4D) and the rupture rate of AAA (Figure 4E) were all more pronounced in GAS5 overexpression construct-transfected mice than in SCR control-transfected mice (p < 0.05). Consistent with these results, overexpression of GAS5 promoted apoptosis in SMCs, as identified by TUNEL assays (Figure 4F-G) and caspase-3 protein levels (Figure 4H-K and Figure S7A) and decreased the number of SMCs (Figure 4L-O and Figure S7B). Therefore, GAS5 upregulation leads to AAA formation by promoting SMC apoptosis.

### Disruption of GAS5 promotes AAA formation in CaCl_2_-treated C57BL/6Jmice

**Figure 5:**
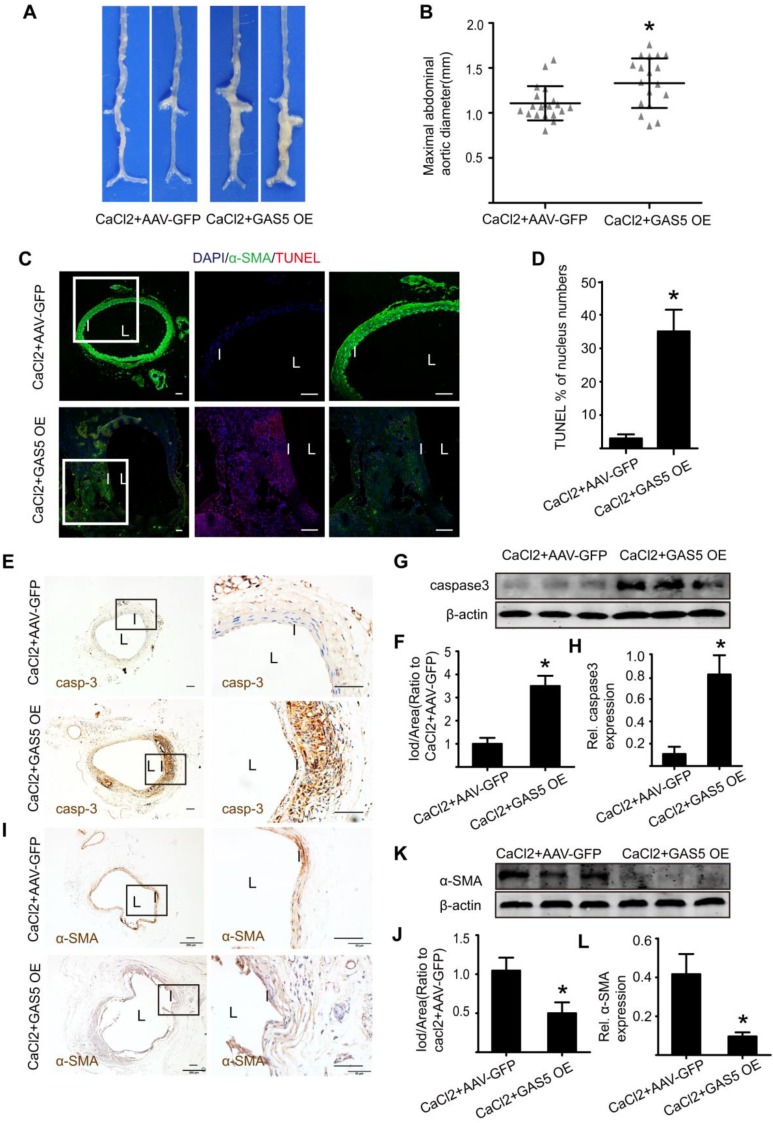
** GAS5 overexpression promotes AAA formation in CaCl_2_-treated C57BL/6J mice**. A. Images depict the characteristics of aortas from C57BL/6J mice treated with CaCl_2_ or CaCl_2_ and GAS5 overexpression constructs. B. Maximal abdominal aortic diameters of C57BL/6J mice treated with CaCl_2_ or CaCl_2_ and GAS5 overexpression constructs. *p < 0.05; n=21 per group (Student's t-test). C. Immunofluorescence staining for DAPI (blue), α-smooth muscle actin (SMA, green) and TUNEL (red) signals in aortas of C57BL/6J mice treated with CaCl_2_ or CaCl_2_ with GAS5 OE constructs (bars: left 200 μm, middle and right 100 μm, magnified images). D. Quantification of TUNEL-positive HASMCs. *P<0.05 vs. SCR control; n= 7 per group (Student's t-test). E. IHC of aortic caspase-3 in CaCl_2_-treated C57BL/6J mice (n=7, bars: left 200 μm, right 50 μm, magnified images) when GAS5 was overexpressed. F. Quantification of caspase-3 protein levels in the aorta (IHC), *p < 0.05; n=7 per group (Student's t-test). G. Caspase-3 protein levels in the aortas of CaCl_2_-treated C57BL/6J mice when GAS5 was overexpressed (western blot) (β-actin internal reference). H. Quantification of caspase-3 protein levels in the aortas of CaCl_2_-treated C57BL/6J mice when GAS5 was overexpressed (western blot). *p < 0.05; n=5 per group (Student's t-test). I. IHC of aortic α-SMA in CaCl_2_-treated C57BL/6J mice (n=7, bars: left 200 μm, right 50 μm, magnified images) when GAS5 was knocked down. J. Quantification of α-SMA levels in the aorta (IHC), *p < 0.05; n=7 per group (Student's t-test). K. α-SMA protein levels in the aortas of CaCl2-treated C57BL/6J mice when GAS5 was overexpressed (western blot) (β-actin internal reference). L. Quantification of α-SMA protein levels in the aortas of CaCl2-treated C57BL/6J mice when GAS5 was overexpressed (western blot). *p < 0.05; n=5 per group (Student's t-test).

To explore whether the modulation of GAS5 on AAA formation is also consistent with other mouse models of AAA, we examined the well-accepted CaCl_2_-induced AAA model in the context of GAS5 overexpression. Three weeks after the CaCl_2_-induced AAA model was established, AAA formation was more obvious in the AAV-GFP-GAS5 group than in the AAV-GFP group (Figure 5A). Consistently, the maximal abdominal aortic diameter was also higher in the AAV-GFP-GAS5 group than in the AAV-GFP group (p < 0.05; Figure 5B). Along with AAA formation, GAS5 overexpression increased SMC apoptosis, as shown by TUNEL (p < 0.05; Figure 5C-D), caspase-3 IHC (Figure 5E-F and Figure S7A) and western blot (Figure 5G-H), and reduced the number of SMCs, as identified by α-SMA IHC (Figure 5I-J and Figure S7B) and western blot (Figure 5K-L). Therefore, the disruption of GAS5 also exacerbates AAA formation and related apoptosis in SMCs in the CaCl_2_-induced mouse AAA model.

### GAS5 regulates HASMC apoptosis through the YBX1/p21 pathway

**Figure 6:**
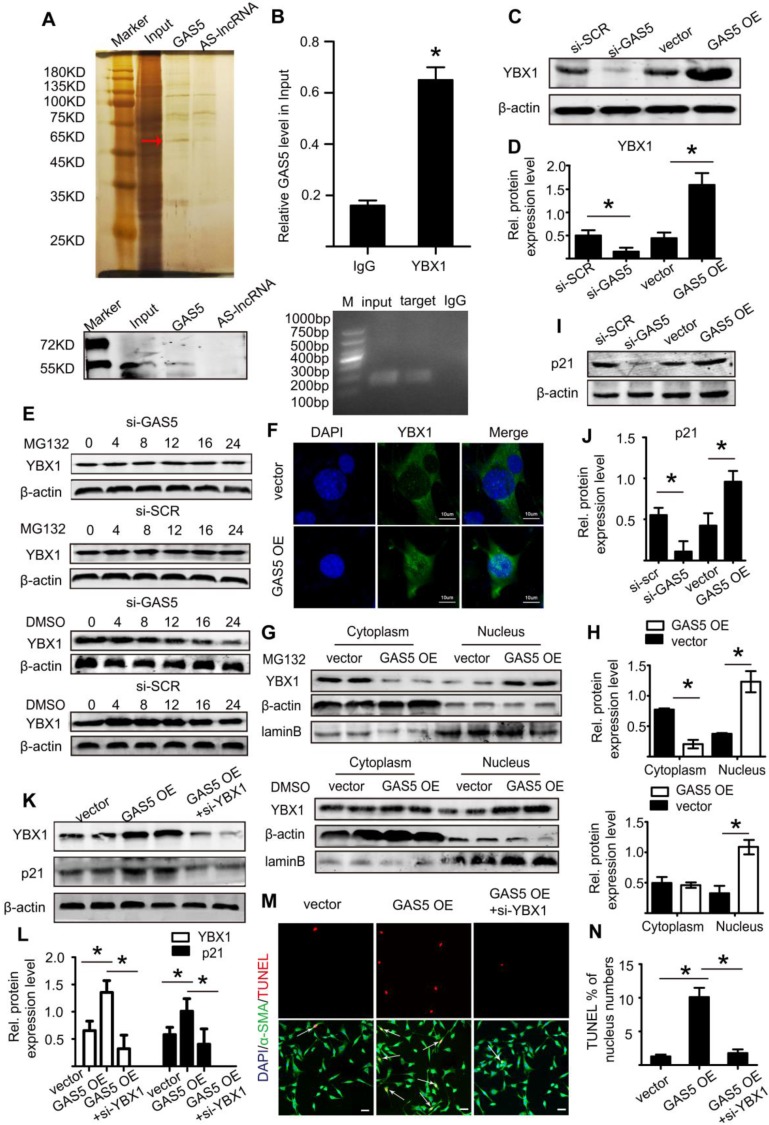
** GAS5 regulates HASMC apoptosis through the YBX1/p21 pathway.** A. Silver-stained sodium dodecyl sulfate polyacrylamide gel electrophoresis gel of proteins immunoprecipitated by GAS5 and its antisense lncRNA. The red arrow indicates the region of the gel that was excised and processed for mass spectrometry.YBX1 protein expression was assayed by western blot. B. RIP experiments were performed using an antibody against YBX1 or negative IgG. *p <0.05 vs. IgG; n= 5 per group (Student's t-test). RIP-derived RNA was measured by qPCR analysis, and GAS5 was expressed as a percentage of input. C. YBX1 protein levels in conditions of GAS5 inhibition or overexpression(western blot) (β-actin internal reference). D. Quantification of YBX1 protein levels when GAS5 was inhibited or overexpressed. *p < 0.05; n=5 per group (one-way ANOVA). E. YBX1 protein levels in HASMCs with or without MG132 treatment at different time points (0, 4, 8, 12, 16, and 24 hours) after transfection with si-GAS5 or si-SCR. F. GAS5 overexpression promoted the entry of YBX1 into the nucleus. G. The distribution of YBX1 in the nucleus and cytoplasm under conditions of GAS5 overexpression with or without M123. H. Quantification of YBX1 protein levels in HASMCs with or without MG132 treatment after transfection with AAV-GAS5 or AAV-empty vector (vector). *p < 0.05; n=5 per group (Student's t-test). I. p21 protein levels under conditions of GAS5 overexpression and knockdown (western blot) (β-actin internal reference). J. Quantification of p21 protein levels when GAS5 was inhibited or overexpressed. *p < 0.05; n=5 per group (one-way ANOVA). K. The protein expression of YBX1 and p21 under the conditions of GAS5 overexpression or GAS5 overexpression and YBX1 knockdown (western blot) (β-actin internal reference). L. Quantification of the YBX1 and p21 protein levels under conditions of GAS5 overexpression or GAS5 overexpression and YBX1 knockdown. *p < 0.05; n=5 per group (one-way ANOVA). M. Immunofluorescence staining for DAPI (blue), α-SMA (green) and TUNEL (red) signals under conditions of GAS5 overexpression or GAS5 overexpression and YBX1 knockdown. N. Quantification of TUNEL-positive HASMCs. *p < 0.05; n=7 per group (one-way ANOVA).

We subsequently explored the mechanism by which GAS5 exerts its roles in HASMCs. We used RNA pull-down assays to determine whether GAS5 functions by interacting with proteins in HASMCs. The bands specific to GAS5 were excised and subjected to mass spectrometry. Among these mass spectrometry-identified proteins, YBX1 was the only protein identified by previous studies that was found to interact with GAS5 (Figure 6A; also see details in Table S9). Furthermore, RIP assays demonstrated that GAS5 was enriched with an anti-YBX1 antibody compared to that achieved with a nonspecific IgG antibody (p < 0.05; Figure 6B), suggesting that GAS5 can bind to YBX1. Like GAS5, the expressions of YBX1 in human and mouse AAA models were all significantly higher than that in normal human and mouse aortas (Figure S8A-L). Next, to clarify whether the association between lncRNA GAS5 and YBX1 affected YBX1 expression, we detected both the mRNA and protein levels of YBX1 when lncRNA GAS5 was knocked down or overexpressed. Interestingly, lncRNA GAS5 affected YBX1 protein levels (p < 0.05; Figure 6C-D), which was further confirmed in Ang II-induced and CaCl2-induced mouse AAA models (p < 0.05; Figure S9A-F). However, GAS5 did not affect YBX1 mRNA levels (Figure S10A), indicating that GAS5 directly interacts with the YBX1 protein rather than the YBX1 mRNA.

We also investigated the molecular consequence of the interaction between GAS5 and YBX1. We first identified that GAS5 could inhibit YBX1 degradation, and this effect could be abolished by the proteasome inhibitor MG132 (Figure 6E), indicating that GAS5 inhibits YBX1 degradation in a proteasome-dependent manner. Then, we found that the interaction between GAS5 and YBX1 led to the entry of YBX1 into the nucleus, as shown by immunofluorescence (Figure 6F). Finally, western blot analysis showed that GAS5 could simultaneously promote the entry of YBX1 into the nucleus while inhibiting YBX1 degradation (p < 0.05; Figure 6G-H), further confirming the abovementioned findings.

YBX1 has been reported to be a transcription factor of the p21 gene and to play a role in the transcription of p21 by entering the nucleus 34. p21, a well-defined protein, plays a similar role as GAS5 and promotes cell apoptosis while inhibiting proliferation 35. We therefore investigated whether GAS5 and YBX1 could regulate the expression of p21. We first constructed three different YBX1-targeting siRNAs to reduce GAS5 expression *in vitro*, of which YBX1 siRNA3 (mediated by siRNA; >73% silencing effect) was the most potent in terms of knockdown efficiency (Figure S10B). Subsequently, we confirmed that YBX1 siRNA3 knocked down YBX1 protein expression (Figure S10C-D). Additionally, knockdown of YBX1 reduced both the mRNA (p < 0.05; Figure S10E) and protein levels of p21 (p < 0.05; Figure S10F-G). Similarly, overexpression of GAS5 promoted the expression of p21, while knockdown of GAS5 had the opposite effect (p < 0.05; Figure 6I-J; Figure S10H). Moreover, the regulatory effect of GAS5 on p21 was inhibited by YBX1 (p < 0.05; Figure 6K-L; Figure S10I), suggesting that this effect of GAS5 on p21 is mediated by YBX1. Furthermore, the proapoptotic effect of GAS5 on HASMCs could be interrupted by YBX1 (p < 0.05; Figure 6M-N). Collectively, these findings suggest that GAS5 regulates HASMC apoptosis through the YBX1/p21 pathway.

### GAS5 regulates HASMC apoptosis through the miR-21/PTEN/Akt pathway

Previous studies have suggested a link between GAS5 and miR-21 in the pathogenesis of cancer 15, osteoarthritis 23, and cardiac fibrosis 21 and shown that miR-21 is a direct target of GAS5. Importantly, miR-21 was identified by a previous study to play a crucial role in AAA formation through the regulation of SMC survival 2. Based on these findings, we hypothesized that GAS5 likely function through miR-21.

**Figure 7:**
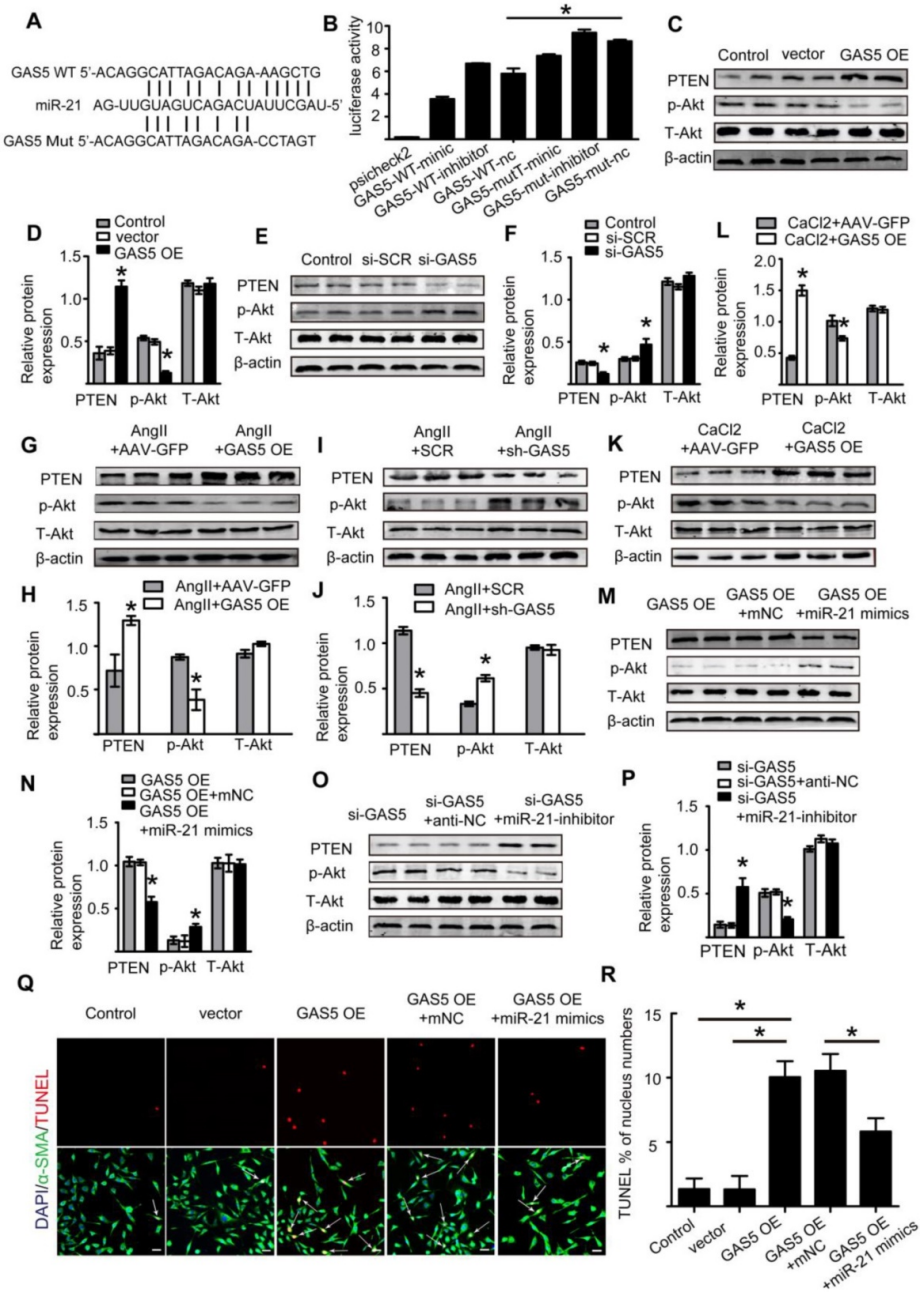
** GAS5 regulates HASMC apoptosis through the miR-21/PTEN/Akt pathway**. A. Potential binding sites of miR-21 on wild-type and mutant GAS5 sequences. B. Results of luciferase reporter gene assays. HASMCs were transfected with miR-21 overexpression constructs or vector and then transfected with a luciferase reporter containing wild-type (GAS5-wt) or mutant GAS5 (GAS5-mut). Luciferase activity was analyzed (mean ± SD). *p < 0.05. C. Expression of the PTEN, p-Akt, and T-Akt proteins in HASMCs when GAS5 was overexpressed (western blot) (β-actin internal reference). D. Quantification of the PTEN, p-Akt, and T-Akt protein levels in HASMCs when GAS5 was overexpressed, *p < 0.05; n=5 per group (one-way ANOVA). E. Expression of the PTEN, p-Akt, and T-Akt proteins in HASMCs when GAS5 was knocked down (western blot) (β-actin internal reference). F. Quantification of the PTEN, p-Akt, and T-Akt protein levels in HASMCs when GAS5 was knocked down, *p < 0.05; n=5 per group (one-way ANOVA). G. Expression of the PTEN, p-Akt, and T-Akt proteins in Ang II-induced mouse AAA tissue when GAS5 was overexpressed (western blot) (β-actin internal reference). H. Quantification of the PTEN, p-Akt, and T-Akt protein levels in Ang II-induced mouse AAA tissue when GAS5 was overexpressed, *p < 0.05; n=5 per group (Student's t-test). I. Expression of the PTEN, p-Akt, and T-Akt proteins in Ang II-induced mouse AAA tissue when GAS5 was knocked down (western blot) (β-actin internal reference). J. Quantification of the PTEN, p-Akt, and T-Akt protein levels in Ang II-induced mouse AAA tissue when GAS5 was knocked down, *p < 0.05; n=5 per group (Student's t-test). K. Expression of the PTEN, p-Akt, and T-Akt proteins in CaCl_2_-induced mouse AAA tissue when GAS5 was overexpressed (western blot) (β-actin internal reference). L. Quantification of the PTEN, p-Akt, and T-Akt protein levels in CaCl_2_-induced mouse AAA tissue when GAS5 was knocked down, *p < 0.05; n=5 per group **(**Student's t-test**)**. M. Expression of the PTEN, p-Akt, and T-Akt proteins in HASMCs when both GAS5 and miR-21 were overexpressed (western blot) (β-actin internal reference). N. Quantification of the PTEN, p-Akt, and T-Akt protein levels in HASMCs when both GAS5 and miR-21 were overexpressed. *p < 0.05; n=5 per group (one-way ANOVA). O. Expression of the PTEN, p-Akt, and T-Akt proteins in HASMCs when both GAS5 and miR-21 were knocked down (western blot) (β-actin internal reference). P. Quantification of the PTEN, p-Akt, and T-Akt protein levels in HASMCs when both GAS5 and miR-21 were knocked down. *p < 0.05; n=5 per group (one-way ANOVA). Q. Immunofluorescence staining for DAPI (blue), α-SMA (green) and TUNEL (red) signals under conditions of GAS5 overexpression or GAS5 overexpression and miR-21 overexpression. R. Quantification of TUNEL-positive HASMCs. *p < 0.05; n=7 per group (one-way ANOVA).

Therefore, using the bioinformatics program RNA hybrid, we found that the seed sequence of miR-21 was complementary to the sequence of GAS5 (Figure 7A). Next, we generated luciferase constructs with wild-type GAS5 (Luc-GAS5-wt) and a mutated form devoid of the miR-21 binding site (Luc-GSA5-mut). MiR-21 could suppress the luciferase activity of Luc-GAS5-wt but had a substantially lower effect on Luc-GAS5-mut (p <0.05; Figure 7B), further indicating that GAS5 directly binds to miR-21. Like GAS5, the expressions of miR-21 in human and mouse AAA were also significantly higher than that in normal human and mouse aortas (Figure S11A-C). GAS5 overexpression reduced miR-21 expression while GAS5 knockdown promotes miR-21 expression in Ang II-induced AAA mouse models (Figure S12A-B). A previous study revealed the key role of the miR-21/PTEN/Akt pathway in AAA development 2. Given these findings and the direct interaction between GAS5 and miR-21 identified by the current study, we speculated that GAS5 regulates the SMC apoptosis by regulating miR-21 and its downstream targets in the PTEN/Akt pathway. Consistently, overexpression of GAS5 promoted the expression of PTEN and thereby inhibited the upregulation of p-Akt (p < 0.05; Figure 7C-D), while knockdown of GAS5 inhibited the expression of PTEN and promoted the downregulation of p-Akt (p < 0.05; Figure 7E-F). The regulatory effects of GAS5 on the PTEN/Akt pathway were further confirmed in Ang II-induced and CaCl2-induced mouse AAA models (p < 0.05; Figure 7G-L).

Subsequently, we constructed a miR-21 inhibitor to reduce miR-21 expression and miR-21 mimics to increase miR-21 expression *in vitro* (Figure S12C-D). Overexpression of GAS5 promoted the expression of miR-21 (Figure S12E), while knockdown of GAS5 inhibited the expression of miR-21 (Figure S12F). Furthermore, the promotional and inhibitory effects of GAS5 on the PTEN/Akt pathway could be abolished by overexpressing or inhibiting the expression of miR-21 (p < 0.05; Figure 7M-P), thus demonstrating that the effect of GAS5 on the downstream PTEN/Akt pathway was mediated by miR-21.We subsequently found that the function of GAS5 in regulating HASMC survival could be interrupted by overexpression of miR-21 (p < 0.05; Figure 7Q-R). As miR-21 has been well established to play a role in AAA formation by regulating SMC survival and our data also show that miRNA-21 promotes HASMC proliferation (Figure S12G-H), the abovementioned findings collectively suggest that GAS5 regulates HASMC apoptosis through the miR-21/PTEN/Akt pathway.

Interestingly, the downstream targets of GAS5, miR-21 and YBX1 did not interact with each other. Knockdown of miR-21 did not affect the expression of YBX1 (Figure S12I-J), and knockdown of YBX1 did not affect the expression of miR-21 (Figure S12K). In order to determine which pathway takes a more important role in GAS5 induced HASMC apoptosis, we overexpressed GAS5, and then knocked down YBX1 and miR21, respectively. We found that YBX1 knockdown decreased apoptosis nearly 2 times as much as that of miR-21 knockdown (Figure S13A-B), indicating that GAS5/YBX1 pathway may be more important than GAS5/miR-21 pathway for GAS5 induced HASMC apoptosis.

### YBX1 enhances the transcription of GAS5 through a feedback mechanism

**Figure 8:**
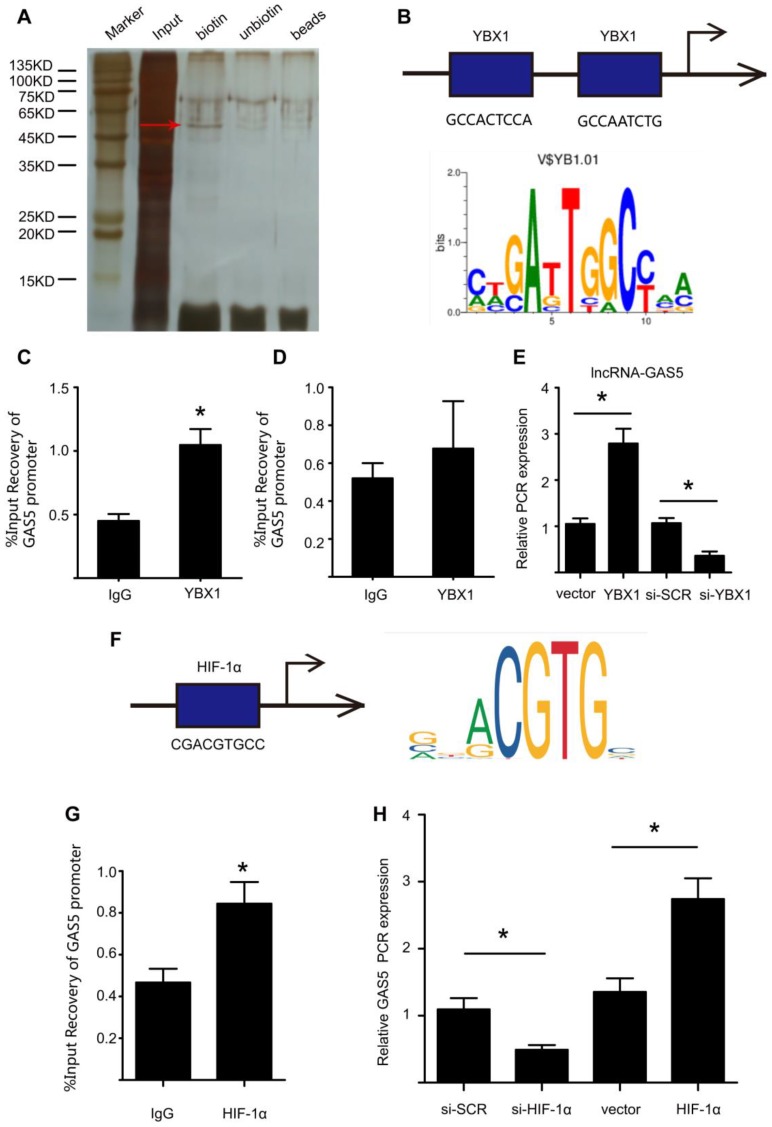
** YBX1 enhances the transcription of GSA5 through a feedback mechanism.** A. Silver-stained sodium dodecyl sulfate polyacrylamide gel electrophoresis gel of proteins immunoprecipitated by the DNA pull-down of biotinylated or unbiotinylated probes targeting GAS5. The red arrow indicates the region of the gel that was excised and processed for mass spectrometry. B. The two predicted YBX1 binding regions and sequences in the promoter region of GAS5. C-D. Chromatin immunoprecipitation experiments were performed using an antibody against YBX1 or negative IgG. Purified RNA was used for real-time qPCR analysis, and enrichment of GAS5 was normalized against the input (n=5 per group). E. The expression of GAS5 when YBX1 was overexpressed or knocked down. *p < 0.05; n=5 per group (one-way ANOVA). F. The predicted HIF-1α binding regions and sequences in the promoter region of GAS5. G. Chromatin immunoprecipitation experiments were performed using an antibody against HIF-1α or negative IgG. Purified RNA was used for real-time qPCR analysis, and enrichment of GAS5 was normalized against the input (n=5 per group). H. The expression of GAS5 when HIF-1α was overexpressed or knocked down. *p < 0.05; n=5 per group (one-way ANOVA).

We further explored the upstream regulatory mechanism of GAS5 by performing a DNA pull-down assay to identify its upstream regulator (Figure 8A; Table S10). Bands specific to GAS5 were excised and subjected to mass spectrometry. Interestingly, YBX1 was also identified as an upstream regulator of GAS5 and could thus regulate the transcription of GAS5. Next, we used bioinformatics analysis to predict the promoter sites to which YBX1 binds. Two promoters were identified (Figure 8B), but only one was demonstrated to bind with YBX1 via chromatin immunoprecipitation (p < 0.05; Figure 8C-D). Furthermore, overexpression of YBX1 promoted the expression of GAS5, while knockdown of YBX1 reduced the expression of GAS5 (p < 0.05; Figure 8E). Therefore, YBX1 acts as both a downstream target of GAS5 and an upstream regulator of GAS5 transcription through a feedback mechanism.

In addition, we predicted possible upstream targets for GAS5 by using the websites of JASPAR (http://jaspar.genereg.net/about/) and ALGGEN (http://alggen.lsi.upc.es/). HIF-1α was found to be the only one possible upstream target that was predicted by both websites. Moreover, HIF-1α has similar functions as GAS5 to promote cell apoptosis. Then, bioinformatics analysis predicted one promoter site to which HIF-1α binds (Figure 8F) and this promoter was demonstrated to bind with HIF-1α via chromatin immunoprecipitation (p < 0.05; Figure 8G). Furthermore, modulation of HIF-1α could regulate the expression of GAS5. HIF-1α overexpression promoted GAS5 expression while HIF-1α knockdown decreased GAS5 expression (Figure 8H). Collectively, these findings indicated that HIF-1α was possible another upstream target that regulated GAS5 expression.

## Discussion

The present study demonstrated that GAS5 plays a crucial role in SMC survival during AAA formation. GAS5 is specifically expressed in the abdominal aorta, and increased GAS5 expression promotes SMC apoptosis while inhibiting proliferation, which subsequently mediates the formation of AAA. Mechanistically, GAS5 participates in AAA formation partly by sponging miR-21 to induce PTEN activity, thereby inhibiting the phosphorylation and activation of Akt. Additionally, GAS5 and YBX1 form a positive feedback loop to regulate downstream p21 expression.

We identified that lncRNA GAS5 plays a functional role in SMC survival during AAA formation. GAS5 regulates SMC survival by not only promoting apoptosis but also by inhibiting proliferation. Moreover, the inhibitory effect of GAS5 on SMC proliferation was robust because GAS5 inhibited SMCs at both interphase (as shown by PH3 expression) and during the overall cell cycle (as shown by Ki-67 expression). As the simple promotion of SMC apoptosis subsequently induced SMC proliferation, the regulation of GAS5 on SMC survival by both apoptosis induction and proliferation inhibition plays a more complementary role in reducing the number of SMCs, a crucial pathological process that causes AAA formation 1. The complementary effects of GAS5 on cell proliferation (inhibitory) and apoptosis (stimulatory) were further supported by data from previous studies performed using multiple cell types 15. Along with shortening SMC survival, GAS5 overexpression induced AAA formation in both Ang II- and CaCl2-induced mouse AAA models. *In vivo*, GAS5 overexpression was associated with increases in the maximal abdominal aortic diameter and rate of AAA incidence, while GAS5 knockdown exerted the opposite effects. Therefore, our results confirmed that GAS5 can regulate SMC survival and subsequent AAA formation. Compared with previous proteins or miR-21 that has been identified to be involved in AAA formation, GAS5 is expressed in an organ-specific manner. Many lncRNAs demonstrate extreme specificity in terms of expression. Consistently, GAS5 was mostly enriched in the aorta, with low expression in other crucial organs/tissues. In addition, lncRNAs often exert their effects in a quantitative manner rather than acting as a switch in genetic regulation, and the side effects of targeting lncRNAs may be easier to control 8. Therefore, the findings that GAS5 regulates SMC survival in AAA formation may provide us with a new strategy against AAA via targeting GAS5, which plays a specific role in preventing AAA, without major side effects on other tissues/organs or with side effects that are easy to control.

In this study, we explored the mechanism underlying the role of GAS5 in AAA formation. To this end, we demonstrated that GAS5 and YBX1 form a positive feedback loop to regulate the downstream target p21. First, the pull-down assay results in our study confirmed that GAS5 can directly bind YBX1, and overexpression or knockdown of GAS5 could correspondingly increase or decrease the expression of YBX1. Consistent with our finding, the regulatory effect of GAS5 on YBX1 was identified by a previous study on stomach cancer 20. In addition, similar to GAS5, YBX1 can promote apoptosis and inhibit proliferation in cells 36. Taken together, these results suggest that GAS5 functions in AAA formation by binding and regulating YBX1. We further showed that p21 was a downstream target of YBX1. Our results demonstrated the positive effect of GAS5 on p21 expression, and this regulation could be abolished by silencing YBX1. Consistent with our findings, p21, a potent cyclin-dependent kinase inhibitor, plays a role similar to that of GAS5 and functions as a regulator of cell cycle progression at the G1 and S phases 35. Moreover, previous studies have identified the regulation of p21 expression and subsequent G1 phase arrest by the YBX1 protein 20, 37. p21 has been shown to be a functional target of GAS5 in ovarian cancer cell lines. Collectively, these findings suggest that GAS5 likely regulates p21 by binding YBX1. Additionally, as a transcription factor, YBX1 functions by binding to the promoters of downstream effector genes and promoting their transcription. In the current study, we also found that GAS5 was regulated by YBX1. We identified a binding site of YBX1 on the GAS5 promoter and demonstrated that YBX1 bound to one of the promoter regions to activate GAS5 transcription. These findings suggest that YBX1 simultaneously acts as an upstream regulator and a downstream target of GAS5. Our study, therefore, uncovered a reciprocal regulatory mechanism between the transcription factor YBX1 and the lncRNA GAS5 that can consequently enhance their mutual promotion of SMC apoptosis during AAA formation.

Accumulating evidence has shown that lncRNAs play various biological roles in disease progression by acting as miRNA sponges. To date, miR-21 is the only miRNA that has been fully demonstrated to play a role in AAA formation 2. Based on these findings, we speculate that GAS5 is an upstream regulator of miR-21 and that it partly regulates AAA formation by suppressing miR-21. We used the RNA22 program to reveal that GAS5 can specifically and directly bind miR-21, and mutations of these binding sites prevent miR-21 from binding GAS5. Consistent with the program prediction, GAS5 overexpression reduced miR-21 expression, while GAS5 knockdown enhanced miR-21 expression. Similarly, previous studies on other diseases, such as cancer 15, osteoarthritis 23, and cardiac fibrosis^21^, have found that GAS5 can act as an miRNA sponge to inhibit miR-21, further supporting the regulatory effect of GAS5 on miR-21. Next, as miR-21 is involved in AAA formation via its suppressive effects on the PTEN/PI3K/Akt signaling pathway, we demonstrated that GAS5 reversed the effects of miR-21, promoted the expression of PTEN and inhibited the phosphorylation and activation of Akt. Moreover, this effect of GAS5 on the PTEN/PI3K/Akt signaling pathway could be abolished by promoting miR-21 expression. Collectively, these data suggest that GAS5 can act as a sponge to bind miR-21 and suppress its downstream signaling pathway.

There are some limitations in the current study. First, GAS5 inhibits the expression of 6-phosphogluconase, an enzyme that plays key roles in glucose metabolism, thereby inhibiting gluconeogenesis and glucolysis 38. Enhanced glycolytic activity in the aortic wall has been demonstrated to contribute to the pathogenesis of aneurysms 39; nevertheless, whether GAS5 contributes to AAA formation through the regulation of glycolytic activity is unclear. Second, in cancer and osteoarthritis, GAS5 has been shown to regulate the expression of MMP2 and MMP9, key enzymes that lead to extracellular matrix degradation, a crucial pathological process involved in AAA formation; however, whether GAS5 promotes extracellular matrix degradation through the regulation of MMP2 and MMP9, thereby contributing to AAA formation, also remains unknown. Third, a previous study demonstrated that GAS5 and miR-21 can both serve as suppressive factors to regulate each other 40, but the current study identified only the inhibitory effects of GAS5 on miR-21. Further investigation is required to determine whether miR-21 also participates in feedback regulation to decrease the expression of GAS5.

In summary, the present study revealed that lncRNA GAS5 is involved in AAA formation through the regulation of SMC survival. GAS5 participates in SMC survival partly by sponging miR-21 to release the suppression of PTEN activity, thereby inhibiting the phosphorylation and activation of Akt. Additionally, GAS5 and YBX1 form a positive feedback loop to regulate downstream p21 expression. Thus, GAS5 may serve as a potential target to prevent AAA formation.

## Supplementary Material

Supplementary figures and tables.

## Abbreviations

LncRNA: long noncoding RNA
AAA: abdominal aortic aneurysm
SMC: smooth muscle cell
Ang II: angiotensin II
YBX1: Y-box-binding protein 1
miR: microRNA
PTEN: phosphatase and tensin homolog
ISH: *in situ* hybridization
HASMC: human aortic smooth muscle cell
IHC: immunohistochemistry
SMA: smooth muscle actin
siRNA: small interfering RNA
SCR: scrambled
PBS: phosphate-buffered saline
shRNA: short hairpin RNA
AAV: adeno-associated virus
qRT-PCR: quantitative real-time PCR
RIP: RNA immunoprecipitation
