# Supplementary material for: Long noncoding RNA GAS5 induces abdominal aortic aneurysm formation by promoting smooth muscle apoptosis
Source: Theranostics. 2019 Jul 28;9(19):5558–76. doi: 10.7150/thno.34463 (PMC6735383; doi:10.7150/thno.34463)
Supplement: Supplementary file 1 — Supplementary figures and tables. [file thnov09p5558s1.pdf]

TableS1

SOP017/05.04

南方医院

南方医科大学南方医院

Medical Ethics committee of NanFang Hospital of Southern Medical University

伦理审查批件 Approval Letter

批件号 No.	NFEC-2019-086		
研究项目名称 Protocol Title	人正常和主动脉夹层/瘤组织基因表达差异		
项目来源 Submitter	南方医科大学南方医院		
研究科室 Research department	心血管内科		
主要研究者 Principal Investigator	宾建平		
审查类别 Scope of review	初始审查 临床科研		
审查方式 Review mode	☐会议审查 Full board review ■快速审查 Expedited review		
审查日期 Review Date	2019 年 05 月 22 日	审查地点 Review Place	NA
审查委员 Review members	孙剑，严金海		
审查文件 Documents	1. 初始审查申请<br>2. 主要研究者简历（最新，签名和日期）临床研究相关培训证明<br>3. GCP 培训证书复印件<br>4. 主要研究者责任声明<br>5. 研究人员职责签名表<br>6. 临床研究方案　（版本号：F2.0，版本日期：2019-04-16）<br>7. 受试者知情同意书　（版本号：Z2.0，版本日期：2019-04-16）<br>8. 质量管理方案<br>9. 项目风险预评估及处置预案<br>10. 合作协议<br>11. 临床研究审批表		
审查意见 Comments	同意		
年度/定期跟踪审查频率 Annual Follow-Up Review Frequency	☐3 个月 3 Months　☐6 个月 6 Months　■12 个月 12 Months		
批件有效期 Expiry of Approval Letter	三年	有效期	2019 年 05 月 22 日至 2022 年 05 月 22 日

主任委员或副主任委员签名：张剑
Signature of the Chair or Vice-chair
南方医科大学南方医院医学伦理委员会（盖章）
Medical Ethics Committee of Nanfang Hospital(Seal)
日期：2019 年 05 月 22 日
Date:

TableS2

南方医院

SOP017/05.04

南方医科大学南方医院

Medical Ethics committee of NanFang Hospital of Southern Medical University

伦理审查意见

意见号	NFEC-201905-K6-修正案审查 01		
研究项目名称	人正常和主动脉夹层/瘤组织基因表达差异		
项目来源	南方医科大学南方医院		
研究科室	心血管内科		
主要研究者	宾建平		
审查类别	修正案审查		
审查方式	☐会议审查　■快速审查		
审查日期	2019 年 06 月 07 日	审查地点	NA
审查委员	孙剑，严金海		
审查文件	1. 修正案审查申请<br>2. 修正说明页以新旧方案对照的表格形式<br>3. 修正的临床研究方案　（版本号：F3.0，版本日期：2019-05-22）<br>4. 知情同意豁免申请　（版本号：H1.0，版本日期：2019-05-22）		
审查意见	同意		
年度定期跟踪审查频率（个月）	12 个月	下次年度定期跟踪审查截止日期	2020 年 05 月 21 日
批件有效期	2019 年 05 月 22 日至 2022 年 05 月 22 日		
主任委员或副主任委员签名：张[illegible]<br>南方医科大学南方医院医学伦理委员会（盖章）<br>日　期：2019 年 06 月 07 日			

联系方式：广州市广州大道北 1838 号，邮编：510515
电话/传真：020-62787238/87713945，邮箱：nfyyec@163.com

Table S3. Patient clinical information（n=10）

patient	gender	age	smoking status	aortic Diameter (mm)	Hyper lipidemia	Hyper tensive	coronary artery disease
ID1	female	72	no	60	no	no	no
ID2	male	69	yes	62	yes	yes	no
ID3	male	71	yes	61	yes	yes	no
ID4	male	61	no	57	yes	no	no
ID5	male	60	yes	68	yes	yes	yes
ID6	male	52	no	60	no	no	no
ID7	male	63	no	56	no	no	no
ID8	male	70	yes	72	yes	yes	yes
ID9	male	68	yes	69	yes	yes	yes
ID10	male	68	yes	70	yes	yes	yes

Table S4. Antibodies for immunohistochemistry analysis

name	Vendor or Source	Catalog #
α-SMA	Abcam	Ab124964
Caspase3	Abcam	ab13847
mmp-2	Abcam	ab37150
mmp-9	Abcam	ab38898

Table S5. Specific siRNAs for depletion or overexpression of GAS5, YBX1, and miR-21, and nonspecific controls

siRNA 1 GAS5,sense: 5’-GCCTAACTCAAGCCATTGG-3’
siRNA 2 GAS5,sense: 5’-GGTATGGAGAGTCGGCTTG-3’
siRNA 3 GAS5,sense: 5’- GCATGCAGCTTACTGCTTG-3’
Human GAS5 variant: NR_002578.3
siRNA 1 YBX1,sense: 5’-CUGCCAUAAAGAAGAAUAATT-3’
siRNA 2 YBX1,sense: 5’-CUGCCAUAAAGAAGAAUAATT-3’
siRNA 3 YBX1,sense: 5’- CGGCAAUGAAGAAGAUAAATT-3’
miR-21 mimic,sense: 5'-UAGCUUAUCAGACUGAUGUUGA-3'
miR-21 inhibitor,sense: 5'-UCAACAUCAGUCUGAUAAGCUA-3'
shRNA GAS5,sense: 5'-GTCGAATGATGTTTAGCATAT-3'
Mouse GAS5 variant: NR_153814.1
shRNA Scramble,sense: 5'-TTCTCCGAACGTGTCACGT-3'

Table S6. Antibodies for immunofluorescent analysis

name	Vendor or Source	Catalog #
Ki-67	Abcam	ab15580
histone H3 phosphorylatedserine10	Abcam	ab47297
α-SMA	Abcam	ab7817

Table S7. Quantitative real-time PCR

	Forward	Reverse
GAS5(human)	TGTGTCCCCAAGGAAGGATG	TCCACACAGTGTAGTCAAGCC
GAS5(mouse)	CACGTGTTCCATCCTGGTCA	GTCAAGGAAGCCCACCATCA
miRNA-21	ACACTCCAGCTGGGTAGCTTATCAGACTGA	TGGTGTCGTGGAGTCG
YBX1	GGACAAGAAGGTCATCGCAAC	TCTCCATCTCCTACACTGCGA
p21	TGTCCGTCAGAACCCATG	TGGGAAGGTAGAGCTTGG
GAPDH(human)	ACGGATTTGGTCGTATTGGG	TGATTTTGGAGGGATCTTGC
GAPDH(mouse)	TGTCCGTCGTGGATCTGAC	CCTGCTTCACCACCTTCTTG

Table S8.Antibodies for western blots

name	Vendor or Source	Catalog #
α-SMA	Abcam	Ab124964
Caspase3	Abcam	ab13847
YBX1	Proteintech	20339-1-AP
p21	Abcam	ab109199
PTEN	Abcam	ab32199
AKT	Cell Signaling Technology	9272
p-AKT	Cell Signaling Technology	4060
β-actin	Bioss	bs-0061R
tubulin	Proteintech	10068-1-AP

Table S9

Majority protein IDs	Protein names	Gene names	Peptides sense1	Unique peptides sense1	Sequence coverage [%]	Unique sequence coverage [%]	Mol. weight [kDa]	Sequence length	Sequence lengths	Q-value	Score	Sequence coverage sense1 [%]	Intensity sense1	LFQ intensity sense1
P67809	elemen t-bindin	YBX1	12	12	43.8	43.8	35.924	324	324;372 ;364	0	110.4 7	43.8	24077	14620
P52597	ogeneo us	HNRN PF	5	4	16.4	14	45.671	415	415	0	38.70 7	16.4	19015	11094
P05455	Lupus La protein	SSB	3	3	8.8	8.8	46.836	408	408	0	24.17 1	8.8	6379.6	3722
Q14137	biogen esis	BOP1	1	1	1.6	1.6	83.629	746	746	0	6.379 4	1.6	4678.8	2729.7
P26641	tion factor 1-gam	EEF1 G	3	3	9.6	9.6	50.118	437	437	0	23.49 8	9.6	2105.5	1228.4
Q12849	sequen ce	GRSF1	2	2	4.2	4.2	53.126	480	480	0	11.75 7	4.2	1001.9	584.52
P06576	se subunit beta	ATP5B	2	2	5.5	5.5	56.559	529	529	0	12.37 1	5.5	656.29	382.9
O60423	transpo rting	ATP8B 3	1	1	0.8	0.8	146.7 5	1300	1300	0	-2	0.8	531.17	309.9
0;P681 04;Q05	3;Elon gation	A1P5; EEF1 A1;EE	5	5	15.4	15.4	50.184	462	462;462 ;463	0	39.25 2	13.6	6493.3	0
Q86U4 2	-bindin g	PABP N1	1	1	8.8	8.8	32.749	306	306	0	17.61 2	5.2	1523.8	0

Table S10

Protein Mass	No. of Peptide	Sequence Header	Relative Abundance	Probability
71317.36	1	>sp|P02768|ALBU_HUMAN Serum albumin OS=Homo sapiens GN=ALB PE=1 SV=2	70.99958%	93.04885%
35902.68	1	>sp|P67809|YBOX1_HUMAN Nuclease-sensitive element-binding protein 1 OS=Homo sapiens GN=YBX1 PE=1 SV=3	29.00042%	89.91142%

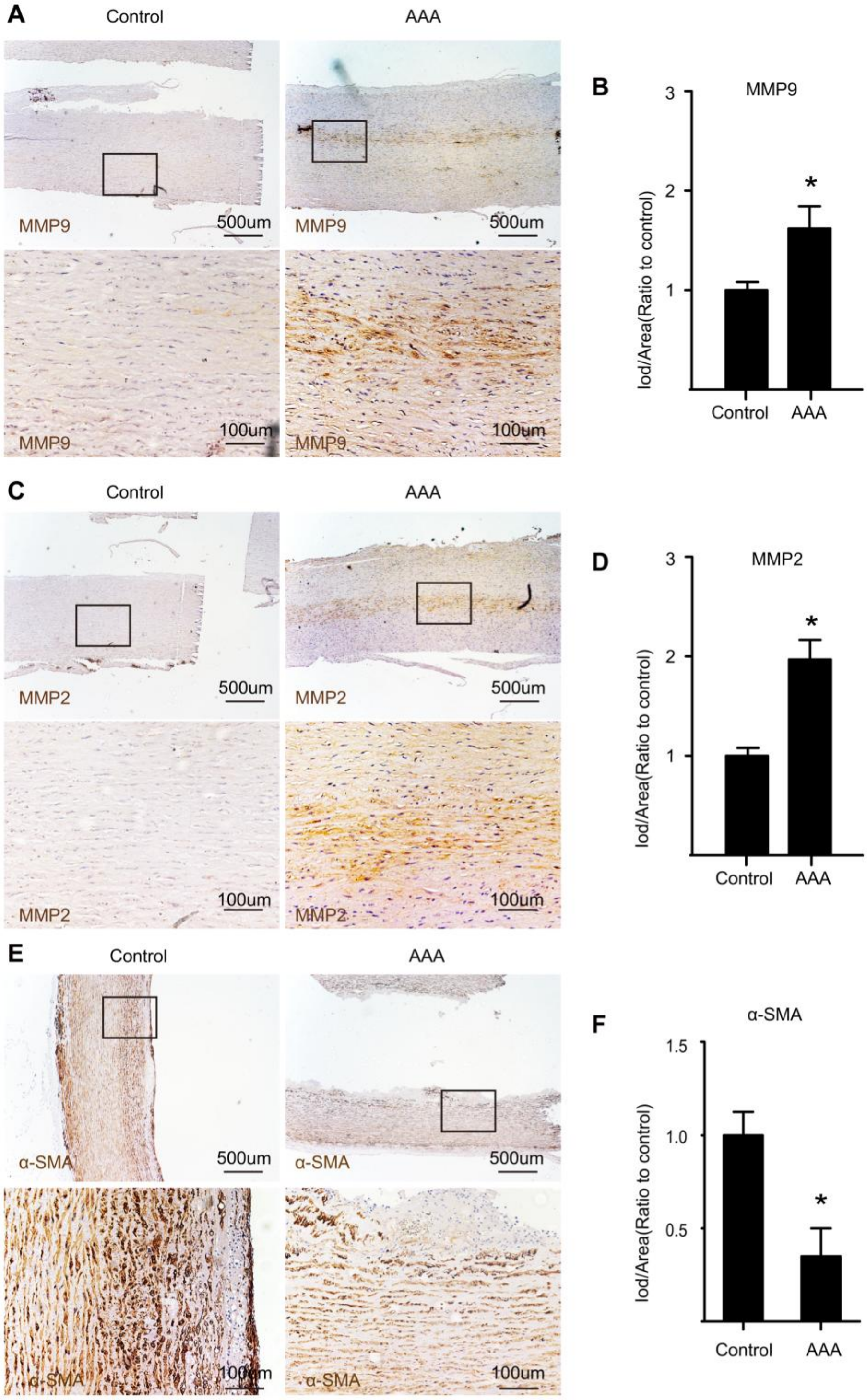

Supplemental Figure 1. A. IHC results of MMP9 in control and human AAA tissues (n = 10, bars: upper 500 μm, lower 100 μm, magnified images). B. The relative expression of MMP9 in control and human AAA tissues (IHC). $*p < 0.05$; n = 10 per group (Student's t-test). C. IHC results of MMP2 in control and human AAA tissues (n = 10, bars: upper 500 μm, lower 100 μm, magnified images). D. The relative expression of MMP2 in control and human AAA tissues (IHC). $*p < 0.05$; n = 10 per group (Student's t-test). E. The relative expression of a-SMA in control and human

AAA tissues (IHC). *p < 0.05; n = 10 per group (Student's t-test). F. IHC results of a-SMA in control and human AAA tissues (n = 10, bars: upper 500 μm, lower 100 μm, magnified images).

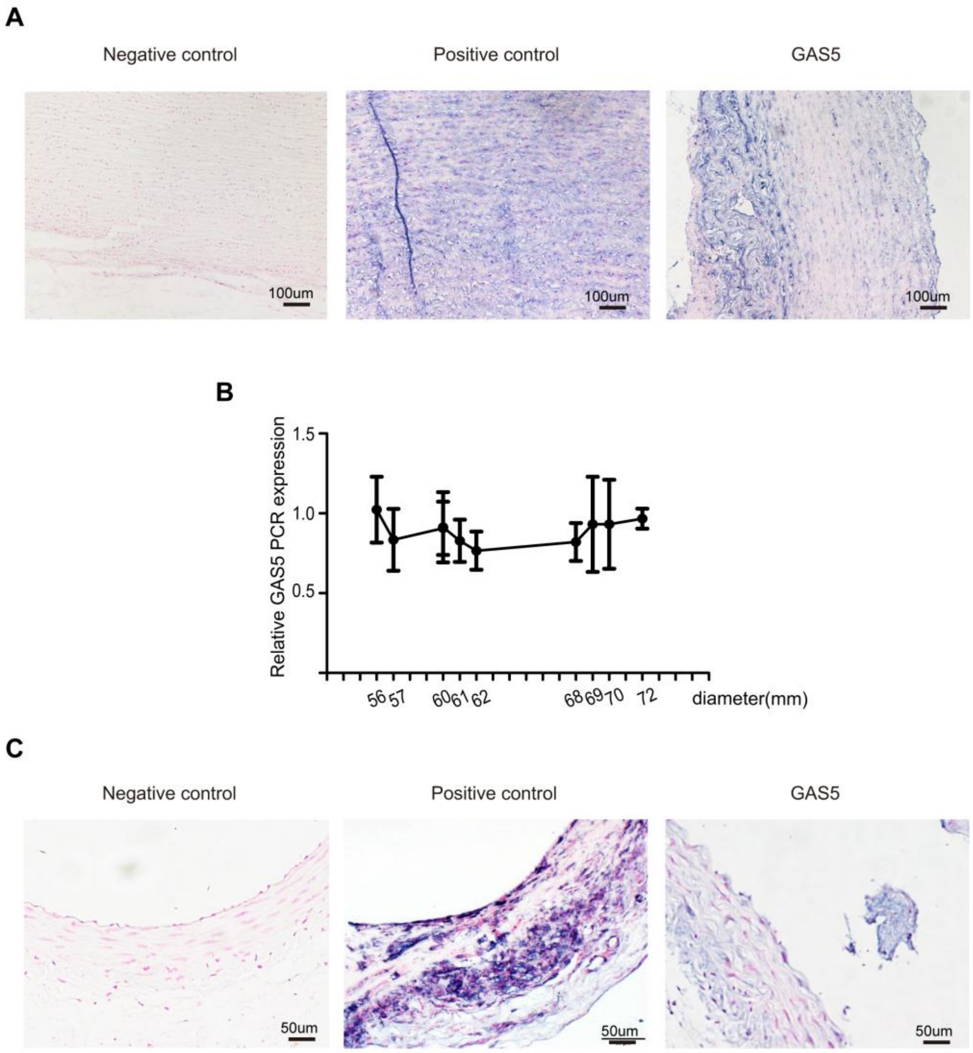

Supplemental Figure 2. A. Negative and positive control experiments confirmed the specificity of the designed probes in the situ hybridization analysis. The transverse sections from the abdominal aneurysm aortas of human are shown (n = 10, bars: 100 μm). B. The association between GAS5 expression and AAA diameter. C. Negative and positive control experiments of ApoE-/- mice tissues confirmed the specificity of the designed probes in the situ hybridization analysis. The transverse sections from the abdominal aneurysm aortas of male ApoE-/- mice are shown (n=5, bars: 50 μm).

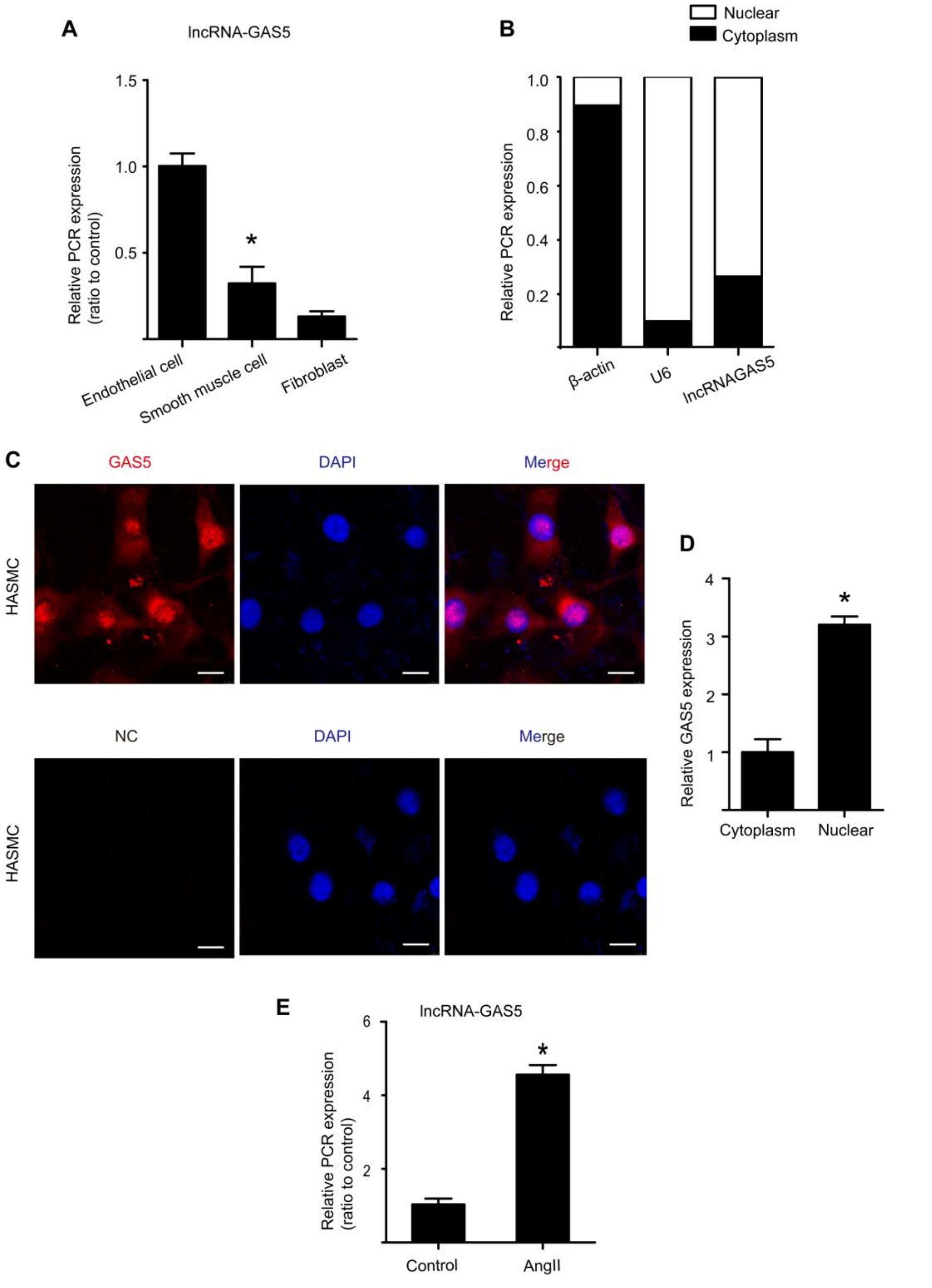

Supplemental Figure 3. A. The relative expression of GAS5 in endothelial cell, smooth muscle cell and fibroblast of human (qPCR). *$p < 0.05$; $n = 5$ per group (one-way ANOVA). B. The relative expression of GAS5 in cytoplasm and nucleus of HASMCs (qPCR). *$p < 0.05$; $n = 5$ per group (student's t-test). C. Representative image of RNA fluorescence in situ hybridization to confirm GAS5 localization in the nucleus (bars, 40 µm). D. Quantification of GAS5 expression in cytoplasm and nuclear. E. The relative expression of GAS5 in HASMCs under conditions with or

without Ang II. *p < 0.05; n = 5 per group (student’s t-test).

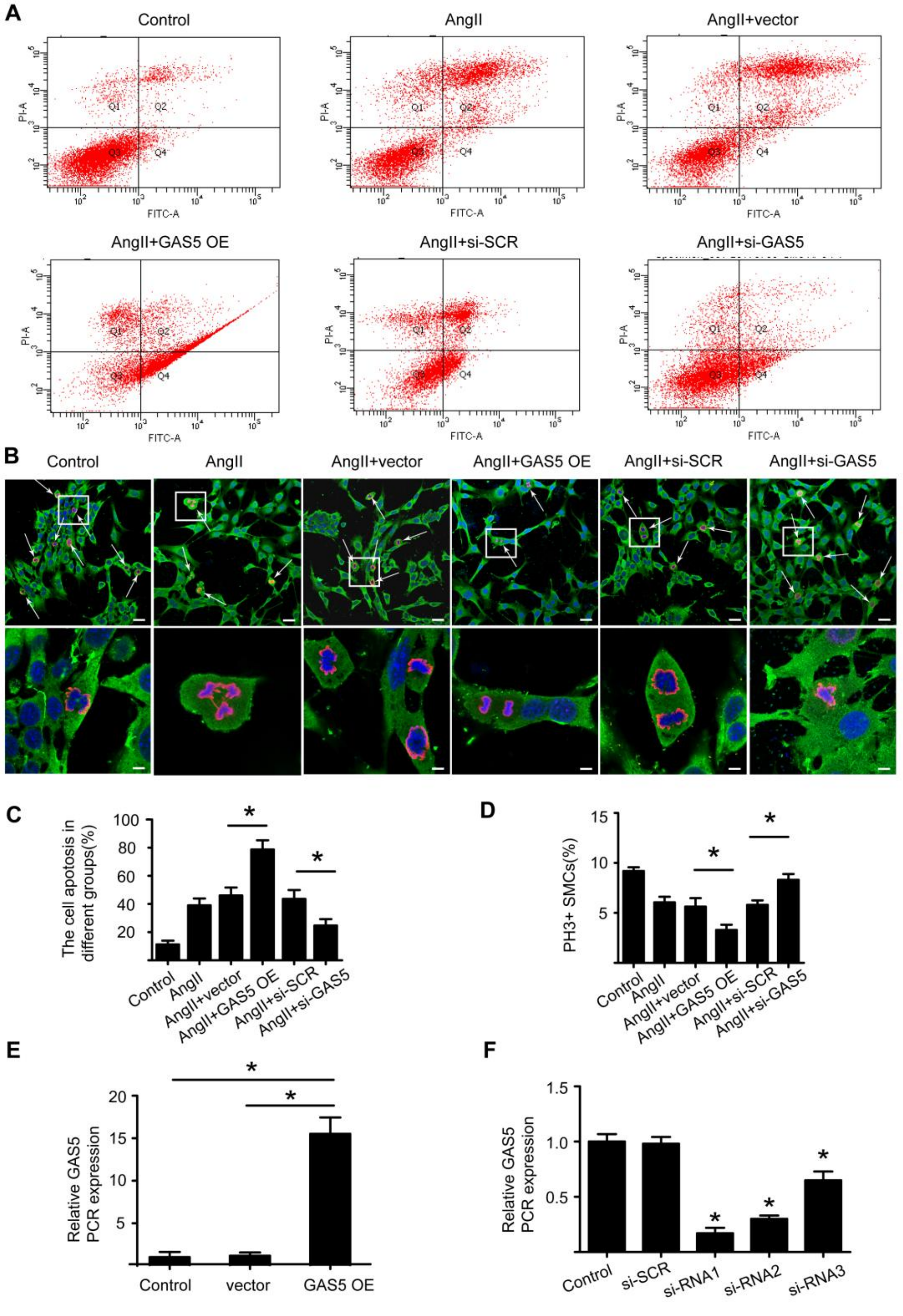

Supplemental Figure 4. A. Results of flow cytometry analysis of HASMCs after treatment with Ang II, Ang II and vector, Ang II and GAS5 overexpression constructs, Ang II and SCR constructs, or Ang II and GAS5 knockdown constructs for 48 hours. B. Immunofluorescence staining for DAPI (blue), α-SMA (green), and PH3 (red) after treatment with Ang II, Ang II and vector, Ang II

and GAS5 overexpression constructs, Ang II and SCR constructs, or Ang II and GAS5 knockdown constructs for 48 hours(bars: upper 100 μm, lower 20 μm, magnified images). C. Analysis of cell apoptosis in different groups. *$p < 0.05$; n =7 per group (one-way ANOVA). D. Quantification of PH3-positive HASMCs. *$p < 0.05$; n=7 per group (one-way ANOVA). E. The expression of GAS5 in HASMCs when GAS5 was overexpressed. *$p < 0.05$ vs control group; n=5 per group (one-way ANOVA). F. The expression of GAS5 in HASMCs after interference with different siRNAs. *$p < 0.05$ vs control group; n=5 per group (one-way ANOVA).

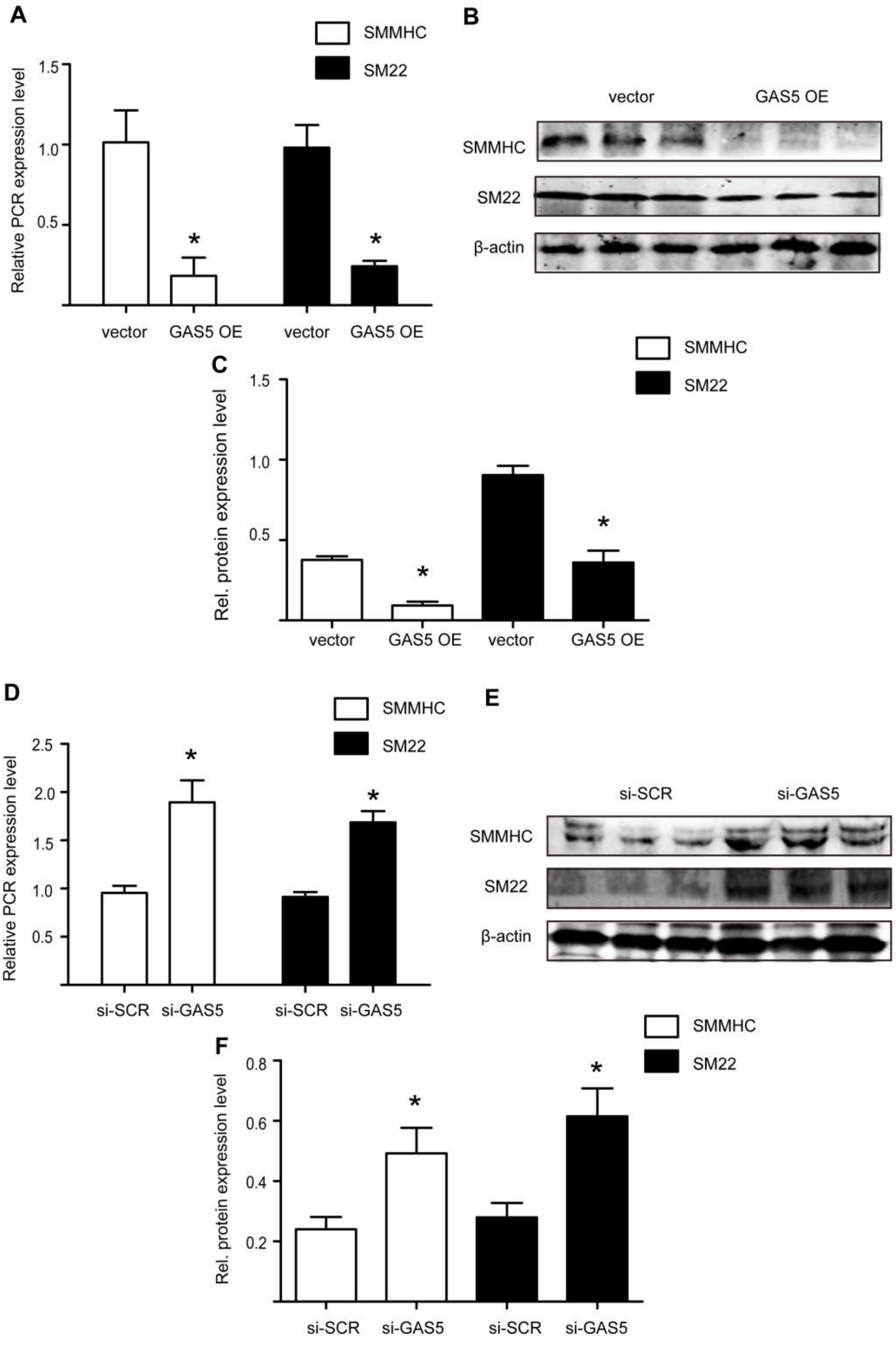

Supplemental Figure 5. A. The relative expression of SMMHC and SM22 in HASMCs when GAS5 was overexpressed (qPCR). *p < 0.05; n = 5 per group (Student's t-test). B. Western blot results of SMMHC and SM22 in HASMCs when GAS5 was overexpressed (β-actin internal reference). C. The relative expression of SMMHC and SM22 in HASMCs when GAS5 was overexpressed (western blot). *p < 0.05; n = 5 per group (Student's t-test). D. The relative expression of SMMHC and SM22 in HASMCs when GAS5 was knocked down (qPCR). *p <

0.05; n = 5 per group (Student's t-test). E. Western blot results of SMMHC and SM22 in HASMCs when GAS5 was knocked down (β-actin internal reference). F. The relative expression of SMMHC and SM22 in HASMCs when GAS5 was knocked down (western blot). *p < 0.05; n = 5 per group (Student's t-test).

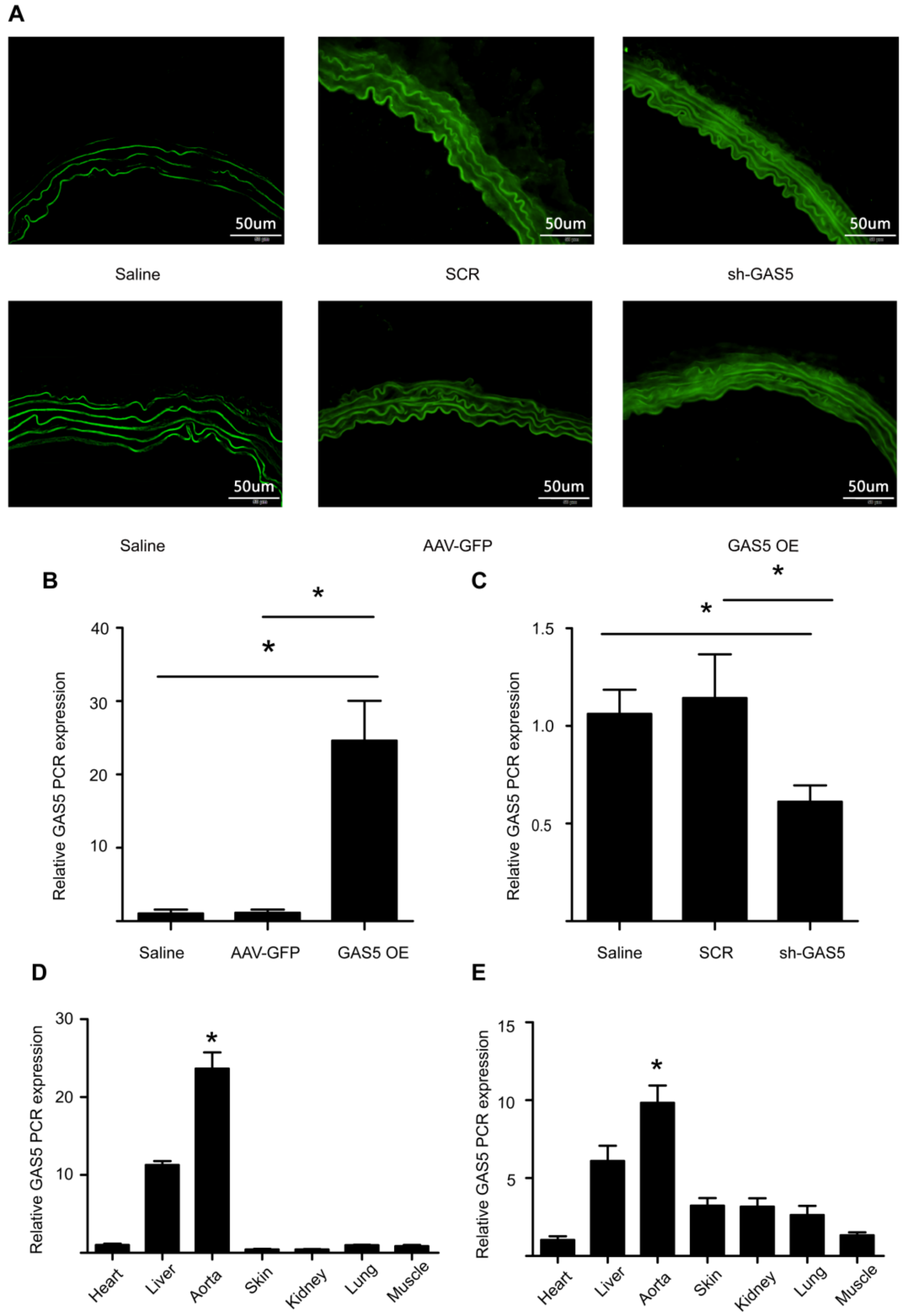

Supplemental Figure 6. A. Representative immunofluorescence staining of virus-borne green

fluorescentprotein (GFP) in the aortas of male mice in different virus-mediated groups and the saline group. B. The expression of GAS5 in aortas when GAS5 was overexpressed. *p < 0.05 vs control group; n=5 per group (one-way ANOVA). C. The expression of GAS5 in aortas when GAS5 was knocked down. *p < 0.05 vs control group; n=5 per group (one-way ANOVA). D. The relative expression of GAS5 in the heart, liver, aorta, skin, kidney, lung, and muscle tissues of C57BL/6J mice when GAS5 was overexpressed (qPCR). *p < 0.05; n = 5 per group (one-way ANOVA). E. The relative expression of GAS5 in the heart, liver, aorta, skin, kidney, lung, and muscle tissues of ApoE-/- mice when GAS5 was knocked down (qPCR). *p < 0.05; n = 5 per group (one-way ANOVA).

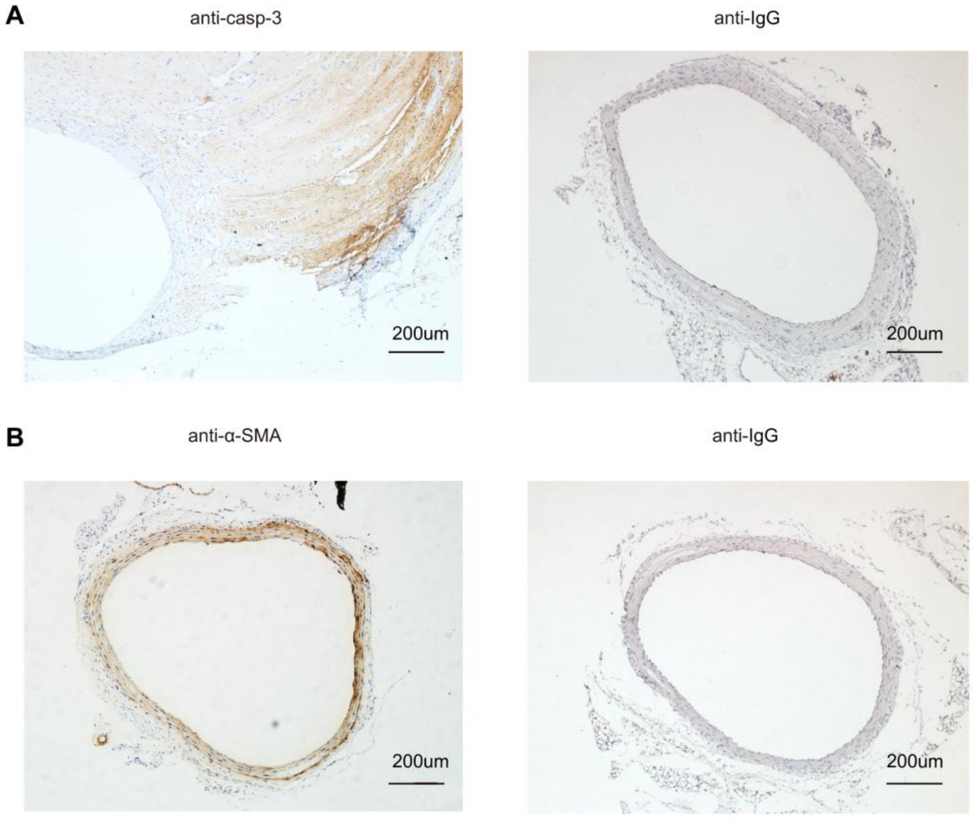

Supplemental Figure 7. Negative control experiments confirmed the specificity of the antibody binding in the immunohistochemistry analysis. A and B, Representative images of immunohistochemical staining for caspase-3 (A) and α-SMA (B). Two transverse sections from the abdominal aneurysm aortas of male ApoE-/- mice are shown (n=5, bars: 200 μm).

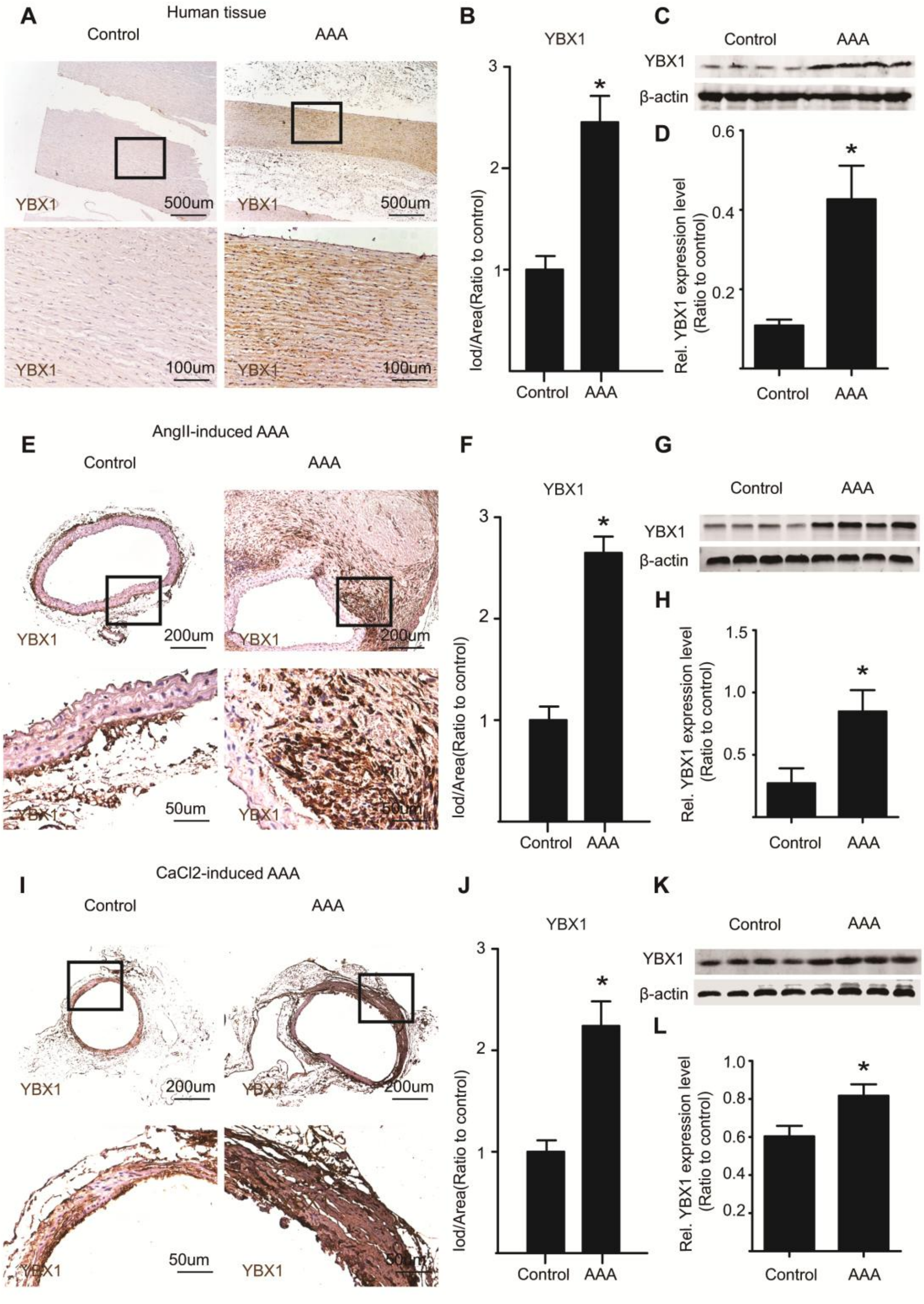

Supplemental Figure 8. A. IHC results of YBX1 in control and human AAA tissues (n=5, bars: upper 500 μm, lower 100 μm, magnified images). B. The relative expression of YBX1 in control and human AAA tissues (IHC). *$p < 0.05$; n = 5 per group (Student's t-test). C. Western blot results of YBX1 in control and human AAA tissues. D. The relative expression of YBX1 in control and human AAA tissues (western blot). *$p < 0.05$; n = 5 per group (Student's t-test). E. IHC results of YBX1 in control tissues and Ang II-induced mouse AAA models (n=5, bars: upper

200 μm, lower 50 μm, magnified images). F. The relative expression of YBX1 in control tissues and Ang II-induced mouse AAA models (IHC). *p < 0.05; n = 5 per group (Student's t-test). G. Western blot results of YBX1 in control tissues and Ang II-induced mouse AAA models. H. The relative expression of YBX1 in control tissues and Ang II-induced mouse AAA models (western blot). *p < 0.05; n = 5 per group (Student's t-test). I. IHC results of YBX1 in control tissues and $CaCl_2$-induced mouse AAA models (n=5, bars: upper 200 μm, lower 50 μm, magnified images). J. The relative expression of YBX1 in control tissues and $CaCl_2$-induced mouse AAA models (IHC). *p < 0.05; n = 5 per group (Student's t-test). K. Western blot results of YBX1 in control tissues and $CaCl_2$-induced mouse AAA models. L. The relative expression of YBX1 in control tissues and $CaCl_2$-induced mouse AAA models (western blot). *p < 0.05; n = 5 per group (Student's t-test).

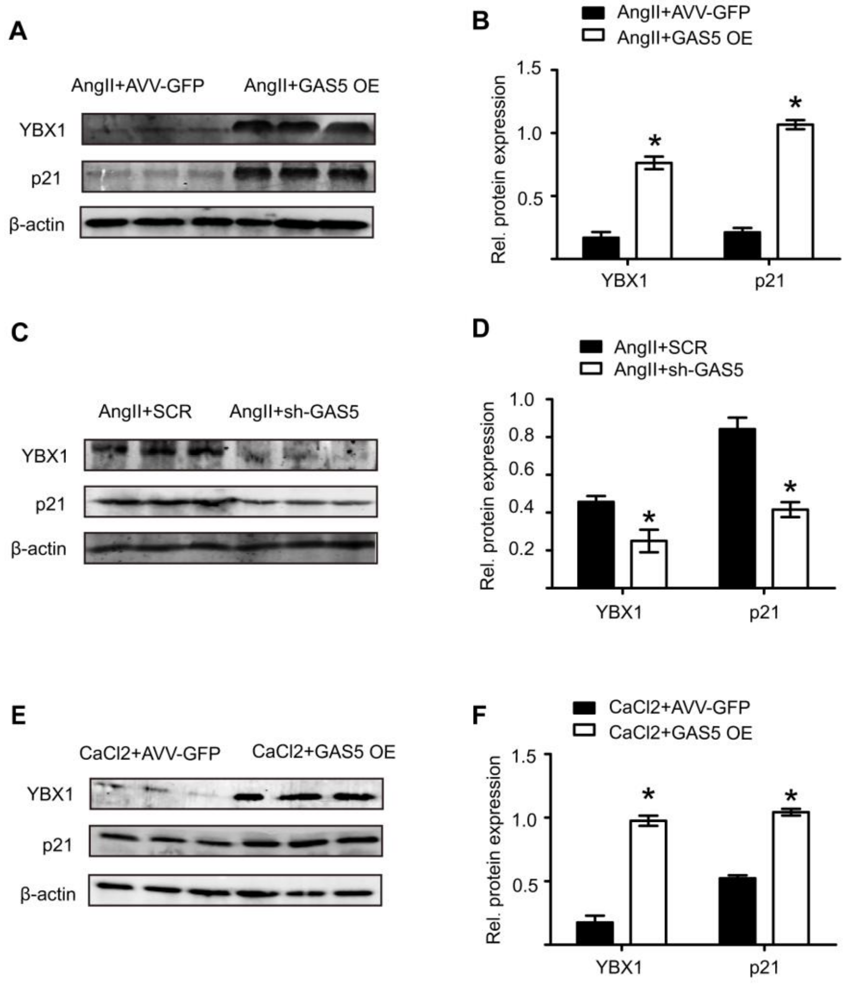

Supplemental Figure 9. A. Expression of the YBX1 and P21 proteins in aortas from C57BL/6J mice treated with Ang II or Ang II with the GAS5 overexpression construct (western blot) (β-actin internal reference). B. Quantification of the YBX1 and P21 protein levels in aortas from C57BL/6J mice treated with Ang II or Ang II with a GAS5 overexpression construct. *p < 0.05; n=5 per group (student's t-test). C. Expression of the YBX1 and P21 proteins in aortas from ApoE-/- mice treated with Ang II or Ang II with a GAS5 knockdown construct (western blot)

(β-actin internal reference). D. Quantification of the YBX1 and P21 protein levels in aortas from ApoE-/- mice treated with Ang II or Ang II with the GAS5 knockdown construct. *p < 0.05; n=5 per group (student's t-test). E. Expression of the YBX1 and P21 proteins in aortas from C57BL/6J mice treated with $CaCl_2$ or CaCl2 with the GAS5 overexpression construct (western blot) (β-actin internal reference). F. Quantification of the YBX1 and P21 proteins in aortas from C57BL/6J mice treated with $CaCl_2$ or CaCl2 with a GAS5 overexpression construct. *p < 0.05; n=5 per group (student's t-test).

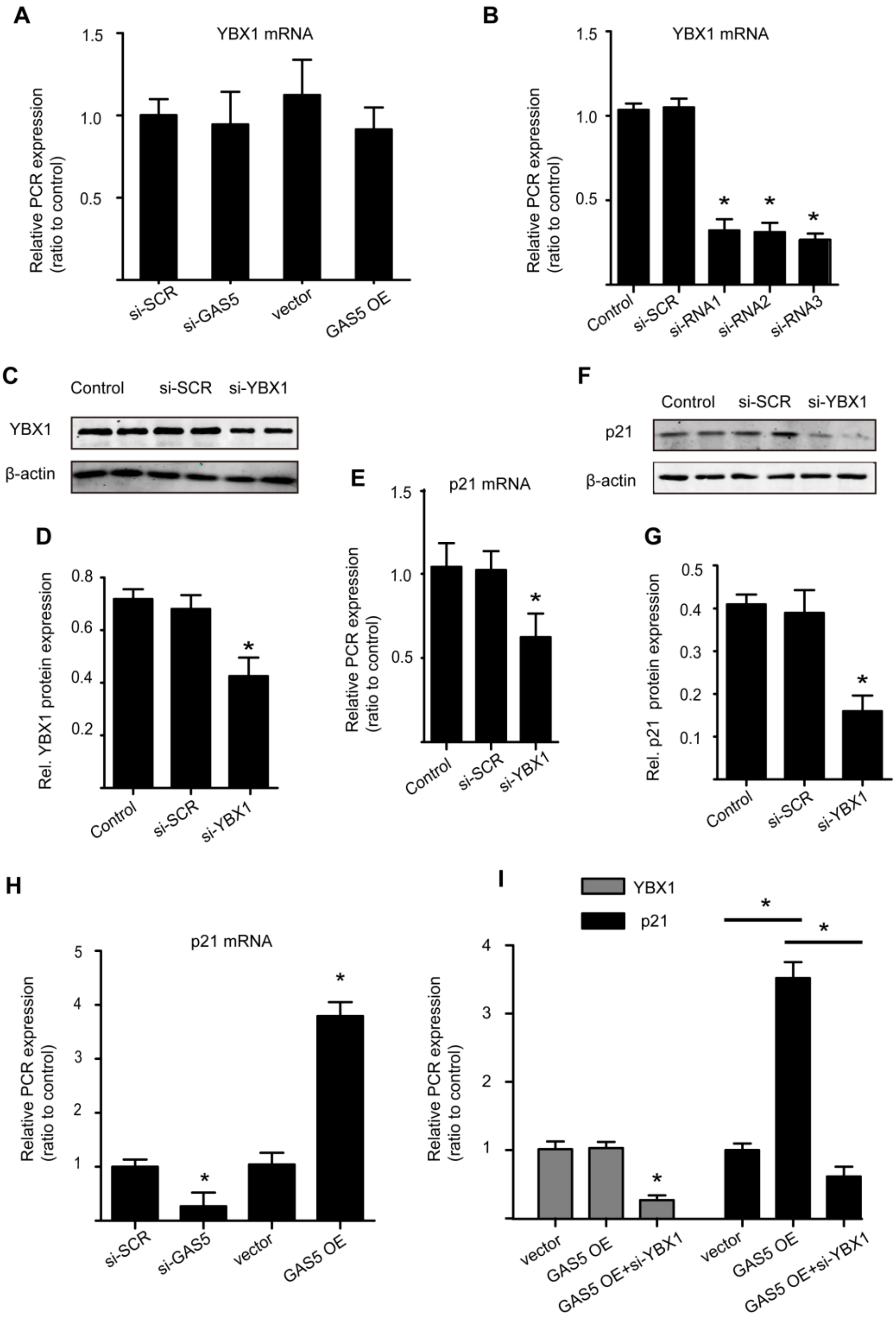

Supplemental Figure 10. A. The mRNA levels of YBX1 in the conditions of GAS5 knockdown or overexpression. B. The mRNA levels of YBX1 in HASMCs after interference with different siRNAs. $*p < 0.05$ vs control group; n=5 per group (one-way ANOVA). C. Expression of the YBX1 protein in HASMCs when YBX1 was knocked down (western blot) (β-actin internal reference). D. Quantification of YBX1 in HASMCs when YBX1 was knocked down. $*p < 0.05$; n=5 per group (one-way ANOVA). E. The mRNA level of P21 when YBX1 was knocked down.

*p < 0.05 vs control group; n=5 per group (one-way ANOVA). F. Expression of the P21 protein in HASMCs when YBX1 was knocked down (western blot) (β-actin internal reference). G. Quantification of P21 in HASMCs when YBX1 was knocked down. *p < 0.05; n=5 per group (one-way ANOVA). H. The mRNA level of P21 when GAS5 was knocked down or overexpressed. *p < 0.05 vs control group; n=5 per group (one-way ANOVA). I. Expression of P21 in conditions of GAS5 overexpression or GAS5 overexpression and YBX1 knockdown. *p < 0.05 vs control group; n=5 per group (one-way ANOVA).

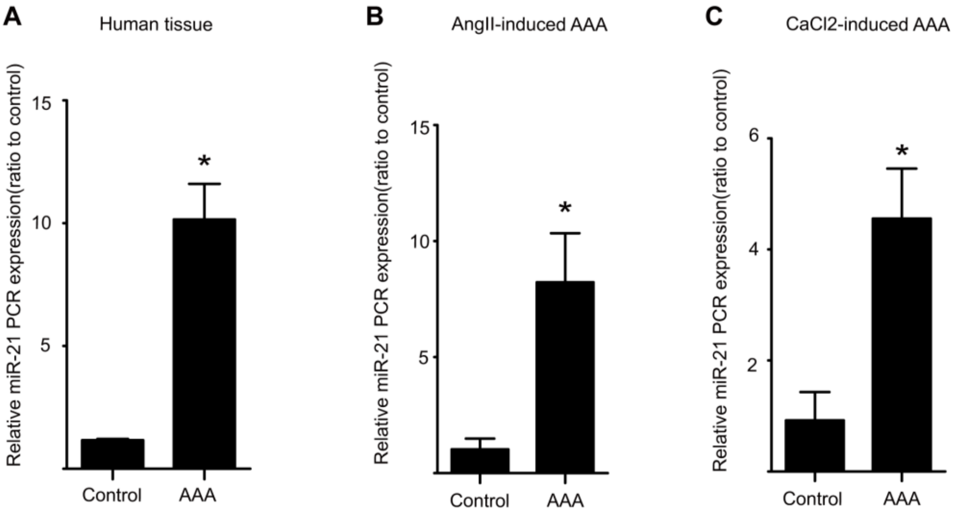

Supplemental Figure 11. A. The relative expression of miR-21 in control and human AAA tissues (qPCR). *p < 0.05; n = 10 per group (student's t-test). B. The relative expression of miR-21 in Ang II-induced mouse AAA models and control tissues (qPCR). *p < 0.05; n = 5 per group (student's t-test). C. The relative expression of miR-21 in $CaCl_2$-induced mouse AAA models and control tissues (qPCR). *p < 0.05; n = 5 per group (student's t-test).

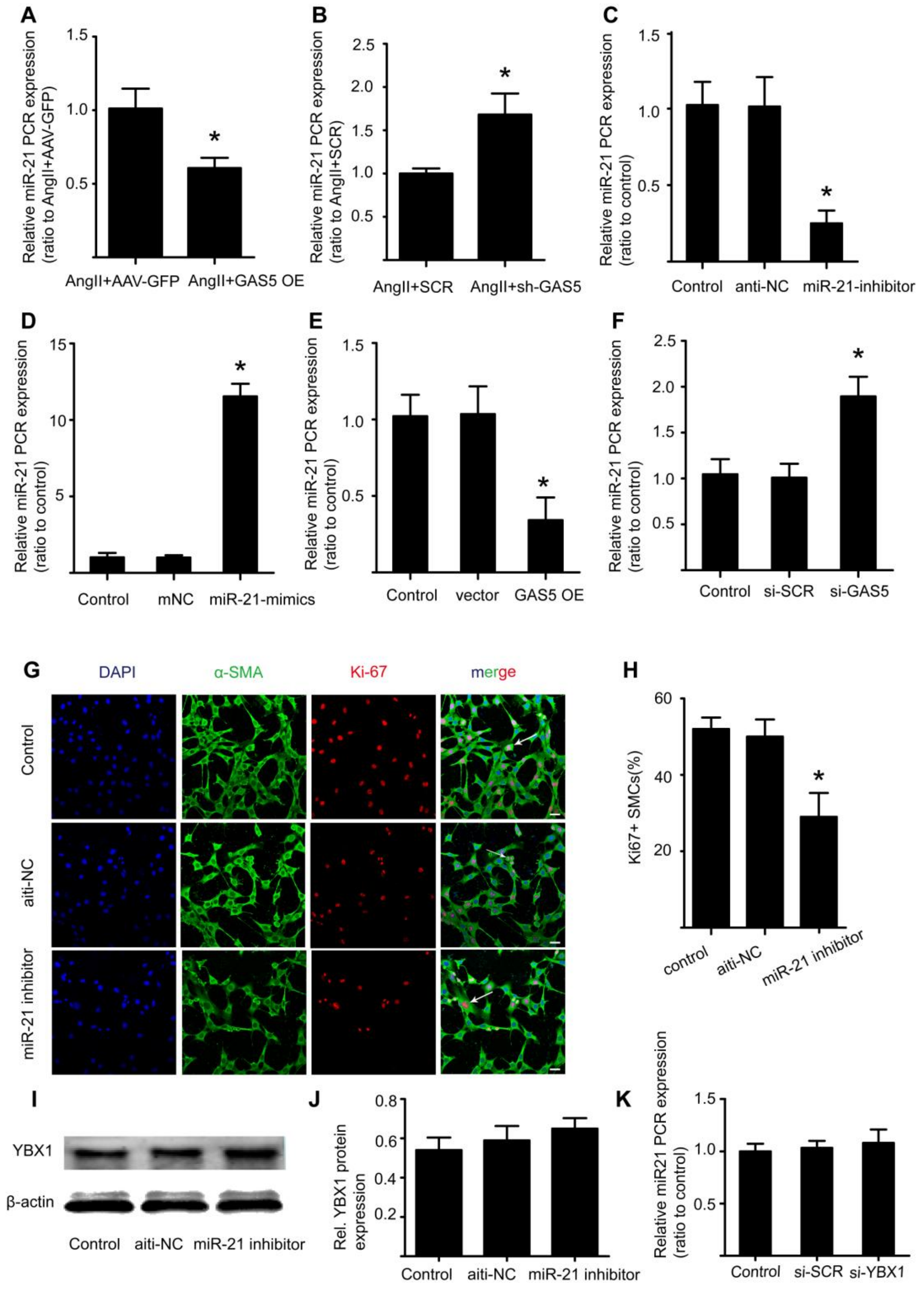

Supplemental Figure 12. A. The miR-21 level in aortas from C57BL/6J mice treated with Ang II or Ang II with the GAS5 overexpression construct. *$p < 0.05$; n = 5 per group (student's t-test). B. The miR-21 level in aortas from AopE-/- mice treated with Ang II or Ang II with the GAS5 knockdown construct. *$p < 0.05$; n = 5 per group (student's t-test). C. The level of miR-21 when miR-21 was inhibited. *$p < 0.05$ vs control group; n=5 per group (one-way ANOVA). D. The level of miR-21 when miR-21 was overexpressed. *$p < 0.05$ vs control group; n=5 per group (one-way

ANOVA). E. The level of miR-21 when GAS5 was overexpressed. *p < 0.05 vs control group; n=5 per group (one-way ANOVA). F. The level of miR-21 when GAS5 was inhibited. *p < 0.05 vs control group; n=5 per group (one-way ANOVA). G. Immunostaining for Ki-67 in HASMCs transfected with miR-21, inhibitor or control(bars, 100 μm). The white arrows refer to Ki-67-positive HASMCs. H. Quantification of Ki-67-positive HASMCs. *P<0.05 vs. control; n=7 per group (one-way ANOVA). I. Expression of the YBX1 protein in HASMCs treated with miR-21, inhibitor or control (western blot) (β-actin internal reference). J. Quantification of the YBX1 protein in HASMCs treated with miR-21, inhibitor or control, *p < 0.05; n=5 per group (one-way ANOVA). K. The level of miR-21 when YBX1 was inhibited. *p < 0.05 vs control group; n=5 per group (one-way ANOVA).

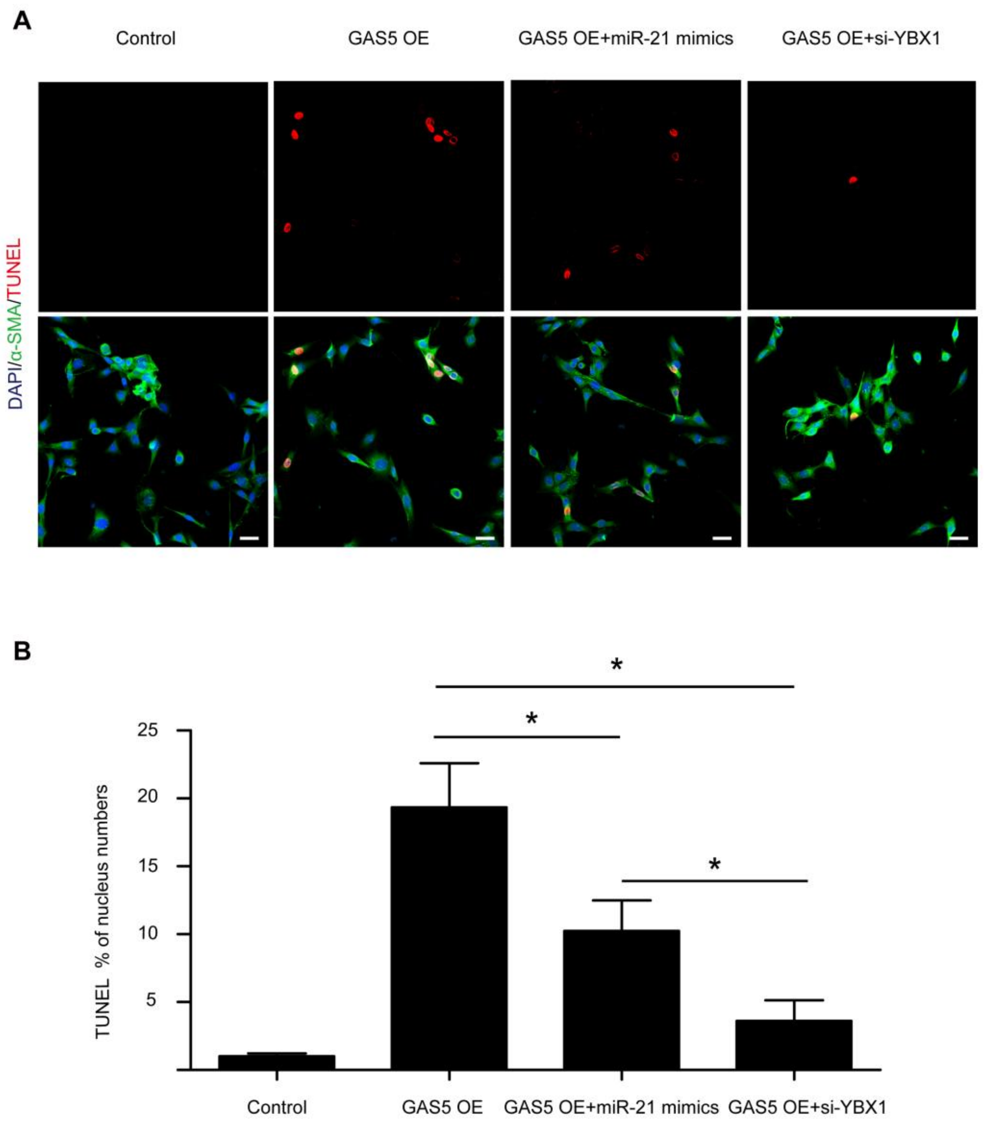

Supplemental Figure 13. A. Immunofluorescence staining for DAPI (blue), α-SMA (green) and TUNEL (red) signals under conditions of GAS5 overexpression, GAS5 overexpression and

miR-21 overexpression, or GAS5 overexpression and YBX1 knockdown. B. Quantification of TUNEL-positive HASMCs. *$p < 0.05$; n=7 per group (one-way ANOVA).
